# A replisome-associated histone H3-H4 chaperone required for epigenetic inheritance

**DOI:** 10.1016/j.cell.2024.07.006

**Published:** 2024-08-01

**Authors:** Juntao Yu, Yujie Zhang, Yimeng Fang, Joao A. Paulo, Dadmehr Yaghoubi, Xu Hua, Gergana Shipkovenska, Takenori Toda, Zhiguo Zhang, Steven P. Gygi, Songtao Jia, Qing Li, Danesh Moazed

**Affiliations:** 1 Howard Hughes Medical Institute, Department of Cell Biology, Harvard Medical School, Boston, USA.; 2 State Key Laboratory of Protein and Plant Gene Research, School of Life Sciences and Peking-Tsinghua Center for Life Sciences, Peking University, Beijing, China.; 3 Department of Biological Sciences, Columbia University, New York, NY 10027, USA.; 4 Department of Cell Biology, Harvard Medical School, Boston, USA.; 5 Institute for Cancer Genetics, Department of Pediatrics, and Department of Genetics and Development, Columbia University Irving Medical Center, New York, NY 10032, USA.; 6 These authors contributed equally.; 7 Lead Contact

## Abstract

Faithful transfer of parental histones to newly replicated daughter DNA strands is critical for inheritance of epigenetic states. Although replication proteins that facilitate parental histone transfer have been identified, how intact histone H3-H4 tetramers travel from the front to the back of the replication fork remains unknown. Here, we use AlphaFold-Multimer structural predictions combined with biochemical and genetic approaches to identify the Mrc1/CLASPIN subunit of the replisome as a histone chaperone. Mrc1 contains a conserved histone binding domain that forms a brace around the H3-H4 tetramer mimicking nucleosomal DNA and H2A-H2B histones, is required for heterochromatin inheritance, and promotes parental histone recycling during replication. We further identify binding sites for the FACT histone chaperone in Swi1/TIMELESS and DNA polymerase α that are required for heterochromatin inheritance. We propose that Mrc1, in concert with FACT acting as a mobile co-chaperone, coordinates the distribution of parental histones to newly replicated DNA.

## In brief

A histone H3-H4 binding domain in the Mrc1/CLASPN component of the replisome is required for parental histone transfer and epigenetic inheritance of heterochromatin. The predicted location of Mrc1 and other histone binding proteins in the replisome suggests a path for parental histone transfer to newly synthesized DNA.

## Graphical Abstract

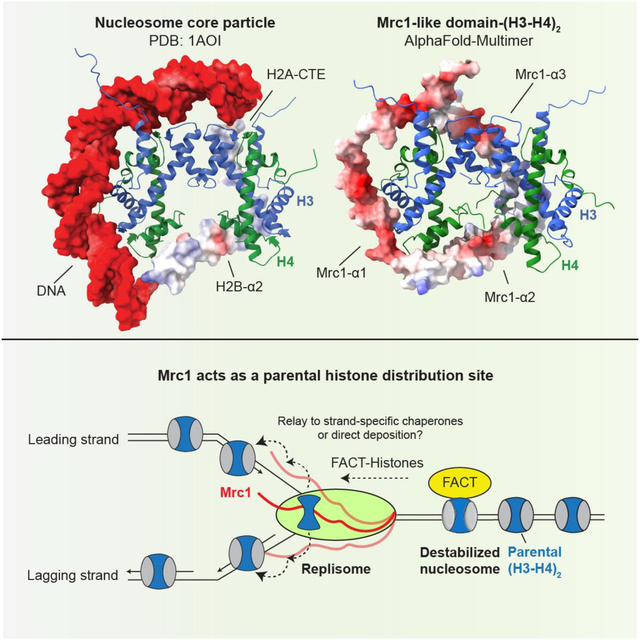

## INTRODUCTION

Cells can maintain a memory of their gene expression programs partly through chromatin-based mechanisms that employ repressive histone modifications^1–6^. Recent studies in the fission yeast *Schizosaccharomyces pombe* have demonstrated that histone H3 lysine 9 trimethylation (H3K9me3), which mediates heterochromatin formation, can be epigenetically inherited independently of DNA sequence^7,8^. The inheritance of H3K9me3 requires the ability of the Clr4/Suv39h methyltransferase to both recognize and catalyze H3K9me3^7–10^. Following DNA replication, this read and write mechanism is thought to copy the methylation on parentally inherited histones onto newly deposited ones to restore H3K9me3 domains and gene silencing. A corollary of this model is that parental histones must be maintained during DNA replication so that the epigenetic information they contain is copied following DNA replication. Indeed, numerous studies have shown that parental histones are retained and transmitted to daughter DNA strands during DNA replication^11–19^. Several genetic screens have identified *S. pombe* replisome components involved in the spreading and maintenance of heterochromatin^20–23^. In addition, histone binding proteins that are either replisome components or replisome-associated histone chaperones have been identified^4,5^. More recent studies have uncovered roles for distinct replisome subunits in preferential transfer of parental histones to the leading versus lagging DNA strands^14–16,19,24^. However, how nucleosomal histones are moved across long distances from the front of the replication fork to the newly synthesized DNA at the back of the fork is not understood.

The nucleosome is composed of 147 base pairs of DNA wrapped around an octameric histone complex containing two H2A-H2B dimers and a core H3-H4 tetramer^25,26^. During DNA replication, H3 and H4 are transferred as an intact tetramer^27^. While H2A-H2B are more dynamic, recent evidence suggests that some modified H2A-H2B are also recycled during DNA replication^19,28^ Multiple replisome components have been shown to bind histone H3-H4, including the Mcm2 subunit of the Cdc45-Mcm2-7-GINS (CMG) replicative helicase^14,15,29–34^, the Pol1 catalytic subunit of the DNA polymerase α^19,24,35^, the Dpb3-Dpb4 subunits of DNA polymerase ε^16,36^, the single strand binding protein complex RPA^37^, and the replication licensing factor Mcm10^38^. Examination of histone association with each newly synthesized DNA strand indicates that distinct replisome components are required for symmetrical distribution of parental histones to the leading and lagging DNA strands^15,16,39,40^. Mutations in the Mcm2 or Pol1 histone binding domains, or mutations that disrupt the coupling of the CMG helicase and DNA polymerase α via Ctf4, lead to preferential histone transfer to the leading strand^14,15,19,24^, whereas deletion of genes encoding Dpb3 or Dpb4 results in biased histone transfer to the lagging strand^16^. Although parental H3-H4 are transferred as intact tetramers^27^, no replisome component that can bind to and stabilize H3-H4 tetramers has yet been identified. In addition to the above proteins, the FACT complex, which has histone H2A-H2B and H3-H4 chaperone activities^41–48^ and can mediate nucleosome retention during transcription elongation^49,50^, is associated with the replisome^51^. FACT is required for efficient replication through chromatin in vitro^52^ and promotes replication-coupled nucleosome assembly^53^, but whether FACT also plays a role in replication-coupled histone transfer remains unknown.

**Figure 1.**
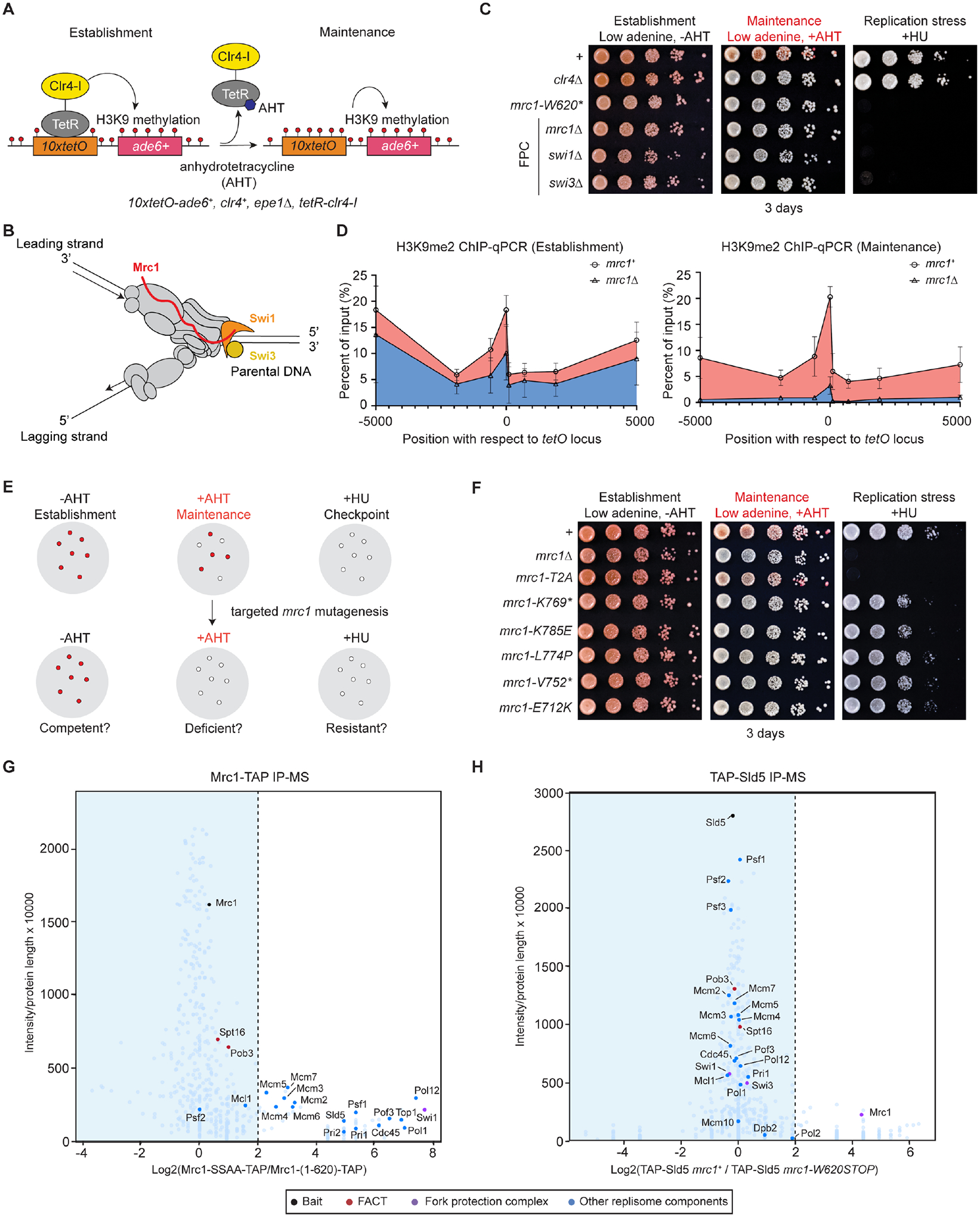
The full fork protection complex is required for heterochromatin maintenance. A) Diagram showing the inducible ectopic heterochromatin system. B) Diagram highlighting the location of the fork protection complex subunits (Swi1, Swi3, Mrc1) on the replisome. C) Heterochromatin maintenance assay testing the roles of subunits of the fork protection complex in epigenetic inheritance. Ten-fold serial dilutions of cells were plated on the indicated growth medium to detect heterochromatin establishment (AHT−) and maintenance (AHT+). Loss of growth on medium containing hydroxyurea (HU+) indicates deficiency in replication checkpoint. * denotes a stop codon. D) H3K9me2 ChIP-qPCR at the *10XtetO-ade6*^+^ locus showing that the H3K9me2 levels in *mrc1*^+^ or *mrc1*Δ cells at the establishment phase (AHT−) and the maintenance phase 24 hours after growth in the presence of AHT. E) Diagram illustrating the gene-targeted random mutagenesis of *mrc1*^+^ to isolate mutant cells that are competent for heterochromatin establishment and replication checkpoint but fail to maintain heterochromatin. F) Separation-of-function alleles isolated from the random mutagenesis of *mrc1*^+^ that abolish heterochromatin maintenance but not replication checkpoint. G) IP-MS analysis of TAP-tagged heterochromatin maintenance-competent Mrc1-SSAA and mutant Mrc1-(1–620). H) IP-MS of TAP-Sld5 in *mrc1*^+^ and *mrc1-W620STOP* cells. G, H) X-axis, the log2 fold change between wild type and mutant epitope tagged proteins; Y-axis, normalized intensity of proteins associated with the indicated tagged proteins detected by mass spectrometry.

We previously used a system for inducible establishment of an ectopic domain of heterochromatin in the fission yeast *S. pombe* to study epigenetic inheritance (Figure 1A)^7,8^. In this system, a *10XtetO-ade6*^+^ reporter gene is inserted at a euchromatic locus and recruits an engineered protein in which the bacterial Tetracycline Repressor (TetR) is fused to the catalytic domain of H3K9 methyltransferase Clr4 (TetR-Clr4ΔCD or TetR-Clr4-initiator, TetR-Clr4-I). The recruitment of TetR-Clr4-I to the *tetO* array results in the formation of ~45 kb H3K9me2/3 domain and silencing of the *ade6*^+^ reporter gene, leading to the formation of red colonies^7^. Growth in the presence of anhydrotetracycline (AHT), which releases TetR-Clr4-I from the *10XtetO* sequence, then allows epigenetic inheritance of heterochromatin to be uncoupled from its sequence-dependent establishment. The separation of heterochromatin establishment and maintenance is particularly useful for uncovering mutations that disrupt parental histone transfer as such mutations would be expected to be specifically defective in heterochromatin maintenance. Using this system, a genome-wide mutagenesis screen identified mutations in several pathways that are specifically required for heterochromatin maintenance, including known heterochromatin-associated factors and the replisome^21^.

In this study, we report on the role of the replisome and its associated histone chaperones in heterochromatin maintenance. Using the inducible heterochromatin system, in combination with biochemical, in vivo, and structural prediction approaches, we identify a role for a conserved component of the replication fork protection complex (FPC), Mrc1/CLASPIN, as a histone H3-H4 tetramer chaperone required for heterochromatin maintenance and efficient recycling of parental histones during DNA replication. We further identify FACT binding sites in the replisome, including in Swi1, another subunit of the FPC, and Pol1, with essential roles in heterochromatin maintenance. AlphaFold-Multimer-guided structural predictions suggest the locations of the histone binding domains of Mrc1 and the FACT complex relative to other histone binding proteins on the replisome. Our findings suggest a model for the transfer of parental H3-H4 tetramers to the newly synthesized leading and lagging DNA strands from an Mrc1 distribution center at the leading edge of the replication fork.

## RESULTS

### Replisome components and histone chaperones required for heterochromatin maintenance

Previous studies have shown that mutations in several replisome components, including Mrc1 and subunits with histone binding activity, have defects in gene silencing^16,21,32,35,54,55^ (Figure S1A–B). However, whether these mutations cause defects in the establishment and/or maintenance of silencing has been unclear. We introduced nonsense mutations in *mrc1* (mrc1-W620*)^21^, or mutations in genes encoding histone binding proteins, *mcm2-3A*^32^*, pol1-6A*^35^*, dpb3*Δ, and *dpb4*Δ in cells carrying the *10XtetO-ade6*^+^ reporter, and found that maintenance of heterochromatin was defective in cells carrying each of the above mutations, suggesting a requirement for Mcm2, Pol1, Dpb3-Dpb4 in heterochromatin maintenance (Figure S1B). Maintenance of heterochromatin did not require the non-essential RPA subunit RFA3 or the alternative clamp loader subunit Ctf18 (Figure S1B), the absence of which was previously shown to have severe synthetic growth defects in combination with *mcm2-3A* in *Saccharomyces cerevisiae*^32^. Consistent with derepression of the *ade6*^+^ reporter gene, ChIP-qPCR experiments showed that in contrast to *mcm2*^+^ cells, H3K9me2 was not maintained in *mcm2-3A* cells 24 hours after the release of the TetR-Clr4-I by growth in AHT-containing medium (Figure S1C–D).

Additionally, several histone chaperones associate with the replisome and may promote replication-coupled chromatin assembly^4,5,56,57^. These include the FACT complex (Spt16, Pob3 and accessory factor Nhp6)^32,52,58–61^, the CAF-1 complex (Pcf1, Pcf2 and Pcf3)^62,63^, Cia1/Asf1^33,34,64^, the SMARCAD family ATPase Fft3^65,66^, and others (Figure S1E). Mutations in genes encoding several of the above proteins have been previously shown to have defects in gene silencing^20,62,65,67–71^. We found that heterochromatin maintenance required the FACT subunit Pob3 and the Fft3 ATPase, but not Nhp6 or subunits of the CAF-1 complex (Figure S1E–F). CAF-1, together with Asf1, which is essential for viability and was not tested here, and other histone chaperones may therefore play more redundant roles in deposition of new histones rather than recycling of parental ones^4,63^. Together, our findings support key roles for Mrc1, FACT, Fft3 and a subset of other replisome components in heterochromatin maintenance.

### Separable roles of Mrc1 in replication checkpoint signaling and epigenetic inheritance

Mrc1 and its metazoan homolog CLASPIN were previously identified as mediators of replication checkpoint signaling^72–74^. Together with two other replication proteins, Swi1/TIMELESS and Swi3/TIPIN, Mrc1/CLASPIN forms the FPC^75–77^ (Figure 1B). We found that, like Mrc1, Swi1 and Swi3 were required for maintenance of heterochromatin (Figure 1C), indicating that the full FPC was required for heterochromatin maintenance. Consistent with its heterochromatin maintenance defects, the H3K9me2 domain at the ectopic locus was not maintained in *mrc1Δ* cells (Figure 1D).

We next tested whether the replication checkpoint function of Mrc1 in resolving replication stress was required for heterochromatin maintenance. When cells encounter replication stress, Mrc1 transduces the stress signal through the hyperphosphorylation of its SQ/TQ motifs to the downstream checkpoint effector kinase Cds1 (Figure S1G,H)^78–80^. In *S. pombe*, two redundant hyperphosphorylated TQs motifs (T645, T653) and one supportive SQ motif (S604) have been identified as the recruitment sites for Cds1 (Figure S1G)^80^. The mrc1-W620* mutation produces a truncated protein that lacks the former SQ/TQ motifs^21^ (Figure S1G). We introduced *mrc1-T645A, mrc1-T653A*, and *mrc1-S604A* single and *mrc1-T645A,T653A (mrc1-T2A)* double amino acid substitutions into cells carrying the *10XtetO-ade6*^+^ reporter. Cells carrying the *mrc1-T2A* mutations became sensitive to HU, similar to *mrc1*Δ cells, indicating sensitivity to replication stress, but were competent in heterochromatin maintenance (Figure S1I). Consistently, heterochromatin maintenance did not require the checkpoint effector Cds1 (Figure S1I), indicating that defects in the replication checkpoint were not responsible for loss of heterochromatin maintenance.

We further performed Taq polymerase-based random mutagenesis of the *mrc1*^+^ gene and isolated additional *mrc1* mutant cells defective in heterochromatin maintenance but competent in transmitting replication checkpoint signals (Figure 1E). We isolated additional *mrc1* missense and nonsense mutations, which localized downstream of the TQ motifs (Figure 1F). Cells carrying these *mrc1* alleles formed white colonies on low adenine medium containing AHT and were resistant to HU, indicating that the C-terminal region of Mrc1 functions in heterochromatin maintenance independently of its replication checkpoint function.

To test whether defective heterochromatin maintenance in *mrc1* mutant cells was due to changes in protein-protein interactions, we performed immunoprecipitation coupled with mass spectrometry (IP-MS) experiments of TAP-tagged Mrc1 proteins. The nonsense mutation at Mrc1-W620 produces a truncated protein that lacks the C-terminal phosphodegron, which stabilizes the mutant protein (Figure S1J)^81^. To generate cells that express similar levels of maintenance-competent and maintenance-defective Mrc1 proteins, we modified the endogenous *mrc1*^+^ gene to express TAP-tagged phosphodegron-deficient Mrc1 (Mrc1-SSAA-TAP, maintenance-competent), and Mrc1-(1–620) (maintenance-defective) for IP-MS analysis (Figure S1J–K). As expected, IP-MS experiments showed that Mrc1 associated with most replisome components (Figure 1G, Table S2)^51^. However, the association of replisome components with truncated Mrc1 was greatly reduced (Figure 1G). We obtained similar results by performing IP-MS from 3xFLAG-tagged *mrc1-SSAA*, *mrc1-W620STOP* (maintenance-defective, checkpoint-defective), or *mrc1-K769STOP* (maintenance-defective, checkpoint-competent) cells (Figure S1L, Table S3). However, in the Mrc1 IP-MS experiment, the spectral counts of the FACT subunit Spt16 and Pob3 were only mildly reduced (Figure 1G; Figure S1L), suggesting that Mrc1 associated with FACT independently of the replisome. In addition, IP-MS analysis of TAP-tagged Sld5, a component of the CMG helicase, from cells expressing Mrc1 or truncated Mrc1-(1–620), supported the observation that association of the truncated Mrc1 protein with the replisome was reduced (Figure 1H). These results raise the possibility that Mrc1 may help transfer parental histones by recruiting a FACT-histone complex or by directly interacting with histones.

### Structural predictions reveal a potential histone H3-H4 tetramer binding interface in Mrc1/CLASPIN

**Figure 2.**
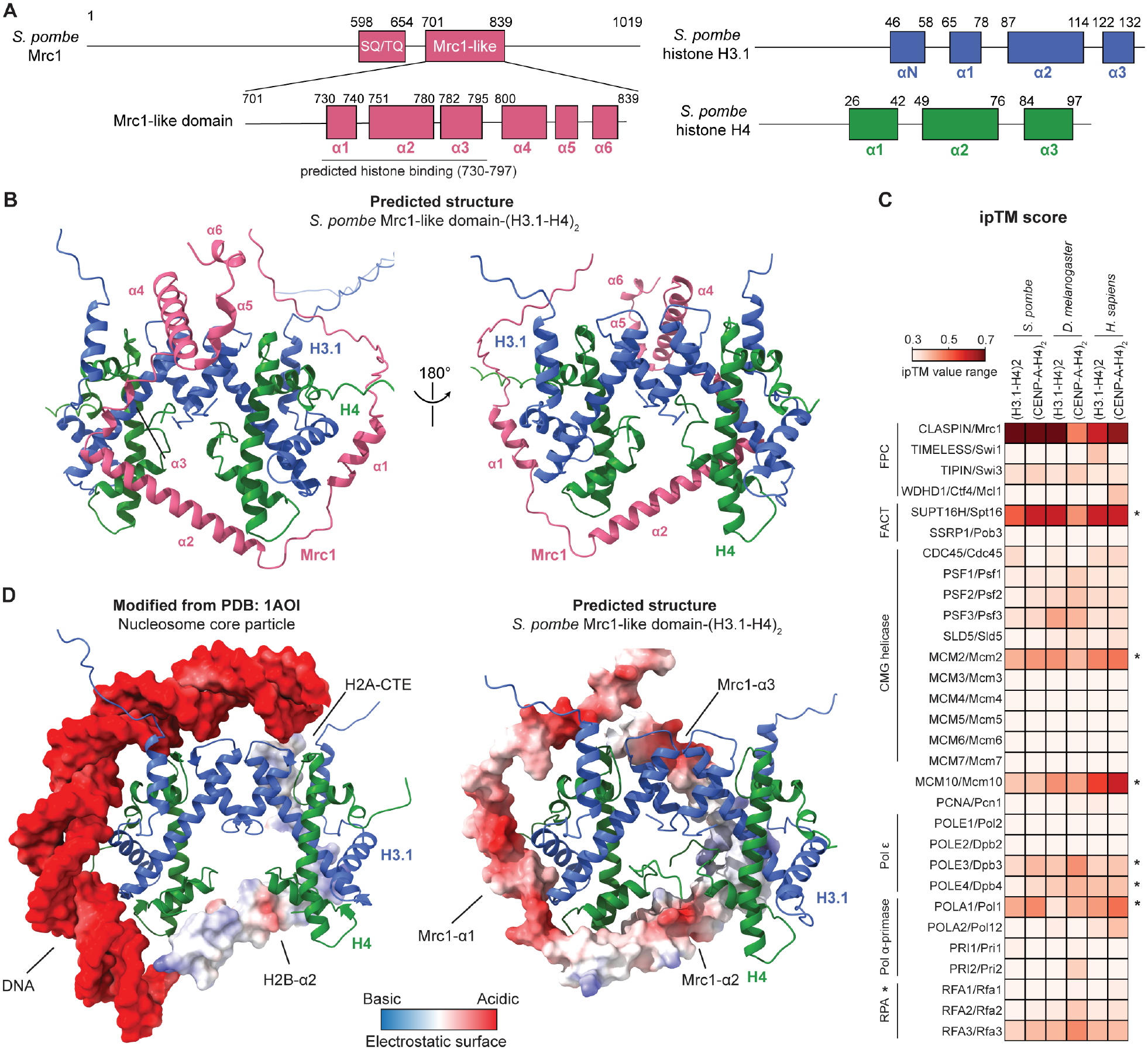
AlphaFold-Multimer predictions suggest an interaction interface between the *S. pombe* Mrc1-like domain and (H3.1-H4)_2_. A) The location of the conserved *S. pombe* Mrc1-like domain and secondary structure features of the Mrc1-like domain predicted by AlphaFold-Multimer. The predicted histone binding domain (amino acid 730 to 797) located within the Mrc1-like domain is indicated at the bottom (left). The structural domains of *S. pombe* histone H3.1 and H4 (right). B) The front (left) and back (right) views of the predicted structure of *S. pombe* Mrc1-like domain-(H3.1-H4)_2_. Mrc1-like domain is colored in pink, and histone H3.1, H4 are colored as blue and green, respectively. C) Heatmap showing the average interface predicted template modeling (ipTM) score of all five predicted models between *S. pombe, D. melanogaster* and *H. sapiens* (H3.1-H4)_2_ or centromere variant (CENP-A-H4)_2_ (X-axis) against each core replisome component (Y-axis). The ipTM score and the heatmap scale range from 0.3 to 0.7. Asterisk denotes known histone chaperones. D) Comparison of the crystal structure of the nucleosome core particle (PDB: 1AOI) (left)^26^ and the predicted structure of Mrc1-like domain-(H3.1-H4)_2_ (right).

Since several replisome components have been shown to bind histones through their unstructured charged regions together with the FACT complex^82^, we asked whether Mrc1 has a histone binding region. Using template-free mode of AlphaFold-Multimer^83–88^, we identified a potential interaction interface between the *S. pombe* Mrc1-like domain (amino acids 701 to 837, Pfam database^89^) and histone H3.1-H4 tetramer with a high confidence score (Figure 2A–B; Figure S2A–B). In the predicted structure, three alpha helices in the Mrc1-like domain (α1–3) form a brace that wraps around a histone H3.1-H4 tetramer in an asymmetric manner (Figure 2B). The fourth to sixth α helices (α4–6) occupied different relative positions in the five predicted models (Figure S2B), suggesting lower confidence in their interaction with the H3.1-H4 tetramer. The α1 and α3 of Mrc1-like domain were predicted to bind each of the two H3-H4 dimers and the intervening α2 helix was predicted to simultaneously interact with both H4 subunits (Figure 2B). This distinctive interaction interface allows a single Mrc1-like domain to bind an entire H3-H4 tetramer, potentially serving to stabilize the H3-H4 tetramer during DNA replication.

To further explore the structural predictions, we performed additional AlphaFold-Multimer predictions and found that i) full length Mrc1 was predicted to interact with H3.1-H4 tetramer as well as centromere variant CENP-A/H4 (Cnp1/H4) tetramer specifically through the predicted Mrc1-like domain (Figure 2C, Figure S2C–F), ii) the predicted Mrc1-histone binding domain was conserved in eukaryotes, and homologs of the Mrc1-histone binding domain from nine major model organisms representing fungi, animals, and plants were predicted to interact with H3.1-H4 tetramers with high confidence scores (Figure S2G), and iii) the interface predicted template modeling (ipTM) score between Mrc1/CLASPIN and H3.1-H4 tetramer, or CENP-A/H4 tetramer from *S. pombe, Drosophila melanogaster* and *Homo sapiens*, were the highest among all replisome components (Figure 2C), including the known histone H3-H4 binding proteins Spt16 and Mcm2, for which experimental structural information is available, and Pol1, Dpb3/Dpb4, and Mcm10, for which no experimental structures are available but AlphaFold predicts relatively high confidence structures (Figure S3A–E). Together, these predictions suggest that Mrc1 contains a conserved histone H3-H4 tetramer binding domain.

We next aligned the predicted Mrc1-like domain-(H3.1-H4)_2_ structure to the crystal structure of the nucleosome core particle (PDB: 1AOI) (Figure S3F, Figure 2D)^26^. The alignment illustrated that the wrapping of the α1 helix of Mrc1-like domain around (H3-H4)_2_ overlaps with nucleosomal DNA (approximately from the dyad to SHL-3, Figure S3G) and the binding of the Mrc1-like domain α2 and α3 helices to (H3-H4)_2_ resembles the interactions of H2B-α2 and H2A C-terminal tail with (H3-H4)_2_ in the nucleosome (Figure 2D; Figure S3G). Compared to H2B-α2, which only interacts with one of the H3-H4 dimer in the nucleosome, α2 of Mrc1-like domain is slightly tilted (~10.855°), permitting it to interact with both H4s in a (H3-H4)_2_ tetramer (Figure S3G). In addition to bearing a structural resemblance, the electrostatic surface of Mrc1-like domain resembles that of nucleosomal DNA, H2B-α2 and H2A’s C-terminal tail (Figure 2D). The Mrc1-like domain therefore may associate with the H3-H4 tetramer in a manner that mimics nucleosome features and leads to partial displacement of nucleosomal DNA and at least one of the two H2A-H2B dimers.

### Experimental validation of the predicted Mrc1 histone binding domain

**Figure 3.**
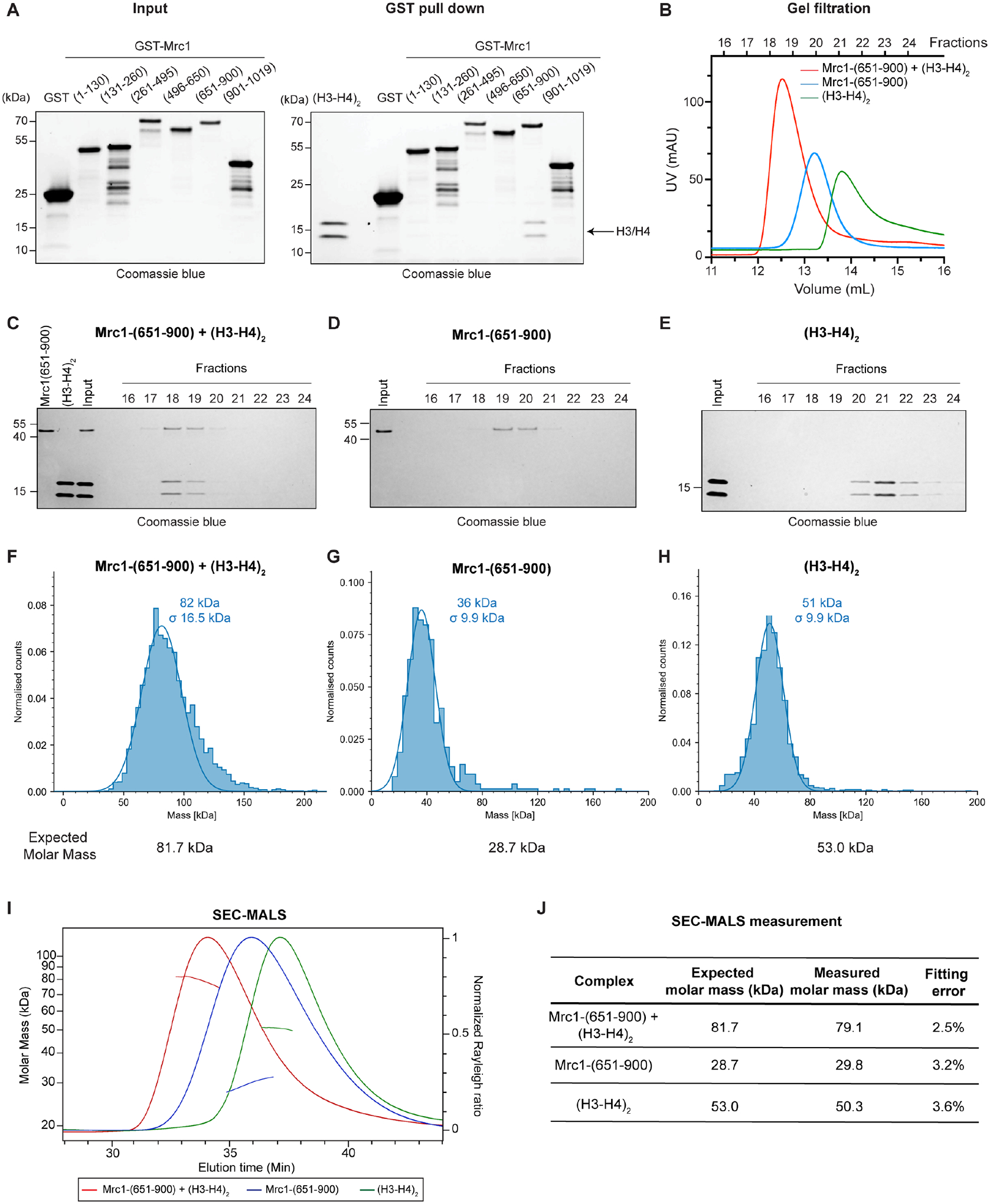
*S. pombe* Mrc1-like domain contains an (H3-H4)_2_ binding domain A) In vitro pulldown assays with GST-Mrc1 fragments immobilized on glutathione magnetic beads and (H3-H4)_2_. B) Chromatogram of purified Mrc1-(651–900), (H3-H4)_2_, and reconstituted Mrc1-(651–900)/(H3-H4)_2_ complex on a Superdex 200 increase 10/300 GL gel filtration column. C-E) SDS-PAGE analysis of peak fractions from the gel filtration column showing comigration of Mrc1(651–900) with H3-H4(C), migration of Mrc1-(651–900)(D), and migration of H3-H4(E). F-H) Mass photometry analysis of the measured molecular mass of purified Mrc1-(651–900)-(H3-H4)_2_ complex(F), Mrc1-(651–900)(G), and (H3-H4)_2_(H). The measurement of Mrc1-(651–900) is higher than the expected molecular weight, which may be due to the detection limit of 30 kDa for mass photometry. I) SEC-MALS profiles of purified Mrc1-(651–900)-(H3-H4)_2_ complex, Mrc1-(651–900), and (H3-H4)_2_. J) Summary of the expected molecular mass and SEC-MALS measured molar mass of purified Mrc1-(651–900)-(H3-H4)_2_ complex, Mrc1-(651–900), and (H3-H4)_2_.

To experimentally test the AlphaFold-Multimer predicted interactions, we performed in vitro pull-down assays using recombinant GST-tagged fragments of Mrc1 to examine their interactions with the histone H3-H4 tetramer. We found that Mrc1 fragment containing the Mrc1-like domain (amino acids 601 to 900), but not other Mrc1 fragments, specifically pulled down histone H3-H4 under stringent binding and wash conditions (500 mM NaCl) (Figure 3A). Consistent with AlphaFold-Multimer predictions, Mrc1-like domain only weakly associated with H2A-H2B (Figure S4A–B). In addition, the Mrc1-like domain of *S. cerevisiae* Mrc1 and human CLASPIN both bound H3-H4, although relative to the human and *S. pombe* Mrc1-like domains, interaction of the *S. cerevisiae* Mrc1-like domain with H3-H4 was more salt-sensitive (Figure S4C–D).

We next reconstituted the *S. pombe* Mrc1-like domain-(H3-H4)_2_ complex using purified Mrc1 fragments without the GST tag and examined the complex using size exclusion chromatography. The Mrc1-(651–900) fragment comigrated with (H3-H4)_2_, at a distinct elution volume relative to free Mrc1-(651–900) or (H3-H4)_2_, suggesting formation of a complex that was stable during chromatography (Figure 3B–E). Mass photometry experiments estimated molecular masses of 82 kDa for the molecules in the peak fraction, close to the expected molecular weight of Mrc1-(651–900)-(H3-H4)_2_ complex (81.7 kDa), 36 kDa for Mrc1-(651–900) (expected 28.7), and 51 kDa for (H3-H4)_2_ (expected 53 kDa) (Figure 3F–H), supporting the predicted structure as a complex of one Mrc1-like domain with one H3-H4 tetramer. Consistent with the mass photometry results, size exclusion chromatography with multi-angle light scattering (SEC-MALS) experiments showed molar masses of 79.1 kDa for the Mrc1-(651–900)-(H3-H4)_2_ complex, 29.8 kDa for Mrc1-(651–900), and 50.3 kDa for (H3-H4)_2_ (Figure 3I–J). We note that the SEC-MALS molar mass of Mrc1-HBD-(H3-H4)_2_ plateaued at 82 kDa (Figure S4E) at the peak front and gradually reduced, suggesting polydispersity in the sample due to disassociation of the complex. The dynamic nature of the Mrc1-HBD-(H3-H4)_2_ interaction may be physiologically important as it would facilitate the transfer of (H3-H4)_2_ to other histone chaperones. Both AlphaFold predictions and biophysical experiments therefore suggest that the Mrc1-like domain associates with a histone H3-H4 tetramer (hereafter referred to as Mrc1-HBD).

**Figure 4.**
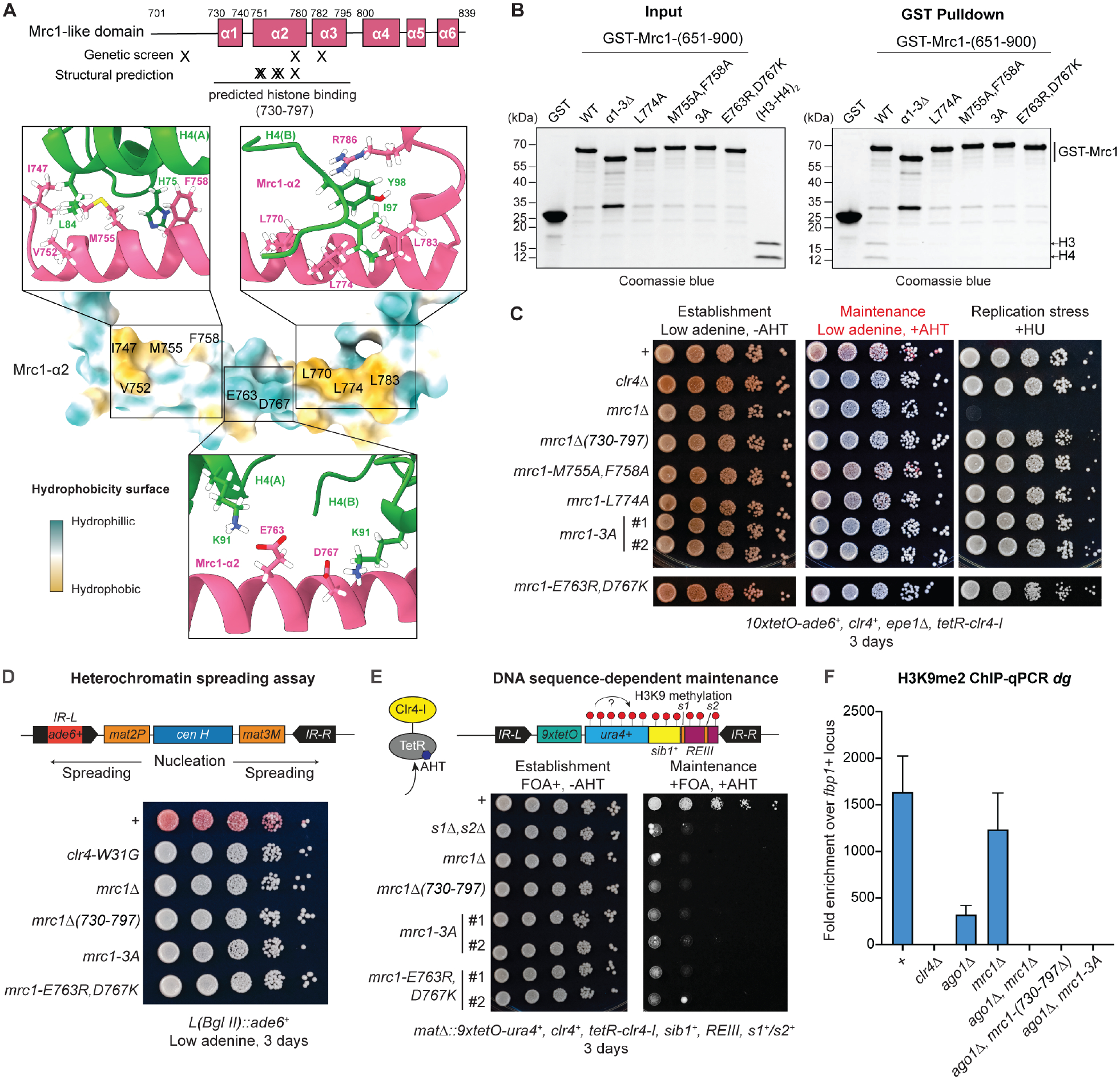
Mrc1 histone binding activity is required for heterochromatin maintenance in *S. pombe*. A) Energy minimized AlphaFold-predicted interaction between Mrc1-α2 and histone H4s. Top, diagram showing the location of Mrc1-α2 and the Mrc1-histone binding domain. Bottom, hydrophobic map of the Mrc1-α2 and detailed predicted interactions between Mrc1-α2 and histone H4. B) In vitro GST pulldown assays showing the effect of hydrophobic (Mrc1-M755A, F758A, L774A) and electrostatic (Mrc1-E763R, D767K) mutations in Mrc1-α2 on histone H3-H4 binding. C) Heterochromatin maintenance assay showing the phenotypes of hydrophobic and electrostatic mutations in *mrc1-α2*. D) Top, diagram showing the *ade6*^+^ reporter gene inserted at the boundary of the mating type locus *IR-L* (*L(BglII)::ade6*^+^). Bottom, silencing assays showing phenotypes of cell carrying Mrc1-histone binding domain mutations in silencing of the *ade6*^+^ reporter. E) Top, diagram showing the DNA sequence-dependent heterochromatin maintenance reporter system in *S. pombe*. Bottom, spotting assay showing the maintenance phenotype of the *ura4*^+^ report gene in wildtype cells and cells carrying the indicated mutations. As a control, cells with deletions of Atf1/Pcr1 binding sites (*s1Δ*,*s2Δ*) are unable to maintain heterochromatin. F) H3K9me2 ChIP-qPCR analysis of *mrc1* mutations in combination of *ago1Δ* at pericentromere *dg* repeats. N=3, error bars indicate standard deviations.

To further test the AlphaFold predictions, we designed point mutations in the Mrc1-HBD, which are predicted to reduce its histone binding activity. The predicted structure suggests that conserved amino acids with acidic side chains (Mrc1-E763,D767) in the middle of Mrc1-HBD α2 helix contact basic residues (H4-K92) in two histone H4s, while the two pockets formed by hydrophobic amino acids at both ends of Mrc1-HBD α2 helix accommodate hydrophobic residues in each of the two histone H4s (Figure 4A). GST pulldown assays under stringent binding conditions showed that mutating several amino acids (M755, F758, L774) in the hydrophobic pockets led to greatly reduced binding of Mrc1-HBD to H3-H4 (Figure 4B). Similarly, substitution of acidic residues in the middle of the Mrc1-HBD α2 helix with basic residues, Mrc1-E763R,D767K, greatly reduced binding to H3-H4 (Figure 4B). Mrc1-HBD α2 acidic and hydrophobic amino acids are therefore required for complex formation with H3-H4.

### The Mrc1 histone binding domain is required for heterochromatin maintenance in S. pombe and S. cerevisiae

Next, we tested the function of Mrc1-HBD in epigenetic inheritance of heterochromatin. We generated *S. pombe* cells that expressed Mrc1 protein lacking the HBD (amino acids 730 to 797, *mrc1-Δ*α*1–3*) and found that the Mrc1-HBD was required for heterochromatin maintenance but not for replication checkpoint signaling (Figure 4C). In addition, we replaced the wild-type *mrc1*^+^ with mutant *mrc1-M755A,F758A, mrc1-L774A, mrc1-M755A,F758A,L774A* (*mrc1-3A*), and *mrc1-E763R,D767K* in cells carrying the *10XtetO-ade6*^+^ reporter. As shown in Figure 4C, in cells carrying the mutant *mrc1-3A*, *mrc1-E763R,D767K*, or the point mutations (*mrc1-E712K, mrc1-L774P*, or mrc1-*K785E*), which map to the Mrc1-HBD domain and were isolated in the genetic screen for heterochromatin maintenance-deficient *mrc1* alleles (Figure 1F; Figure S4F), heterochromatin maintenance was abolished (Figure 4C). However, *mrc1-L774A* single and *mrc1-M755A,F758A* double mutant cells had only weak heterochromatin maintenance defects (Figure 4C, Figure S4G), suggesting that their defective H3-H4 binding under stringent in vitro binding conditions can be partially compensated in the context of full-length Mrc1 and the replisome in vivo.

Cells expressing Mrc1 protein lacking the HBD (*mrc1-ΔHBD*, amino acid 730–797) or mutant Mrc1-3A, Mrc1-E763R,D767K proteins, were also defective in heterochromatin spreading and DNA sequence-dependent epigenetic inheritance at the endogenous *S. pombe* mating type locus (Figure 4D–E)^90–92^. At the pericentromeric DNA repeats, heterochromatin is continuously established by the RNAi pathway^93,94^. Deletion of *mrc1*^+^ by itself had only a minor effect on H3K9me2 levels, suggesting that Mrc1 was not required for RNAi-dependent establishment of H3K9me2 (Figure 4F). In the absence of RNAi, residual H3K9me at pericentromeric repeats is epigenetically maintained by a Clr4 read/write-dependent mechanism^7^. Combining a deletion of *ago1*^+^ (*ago1*Δ) with deletion of *mrc1*^+^, or *mrc1-HBD*, or *mrc1-3A* (*ago1*Δ, *mrc1*Δ; *ago1*Δ, *mrc1*Δ*HBD;* *ago1*Δ, *mrc1-3A*) abolished the residual H3K9me2 (Figure 4F), indicating that Mrc1-HBD was required for epigenetic inheritance of pericentromeric H3K9me2. Together, these observations provide independent support for the structural predictions and further demonstrate that the Mrc1 histone binding domain plays an important role in maintenance of native heterochromatin in *S. pombe*.

**Figure 5.**
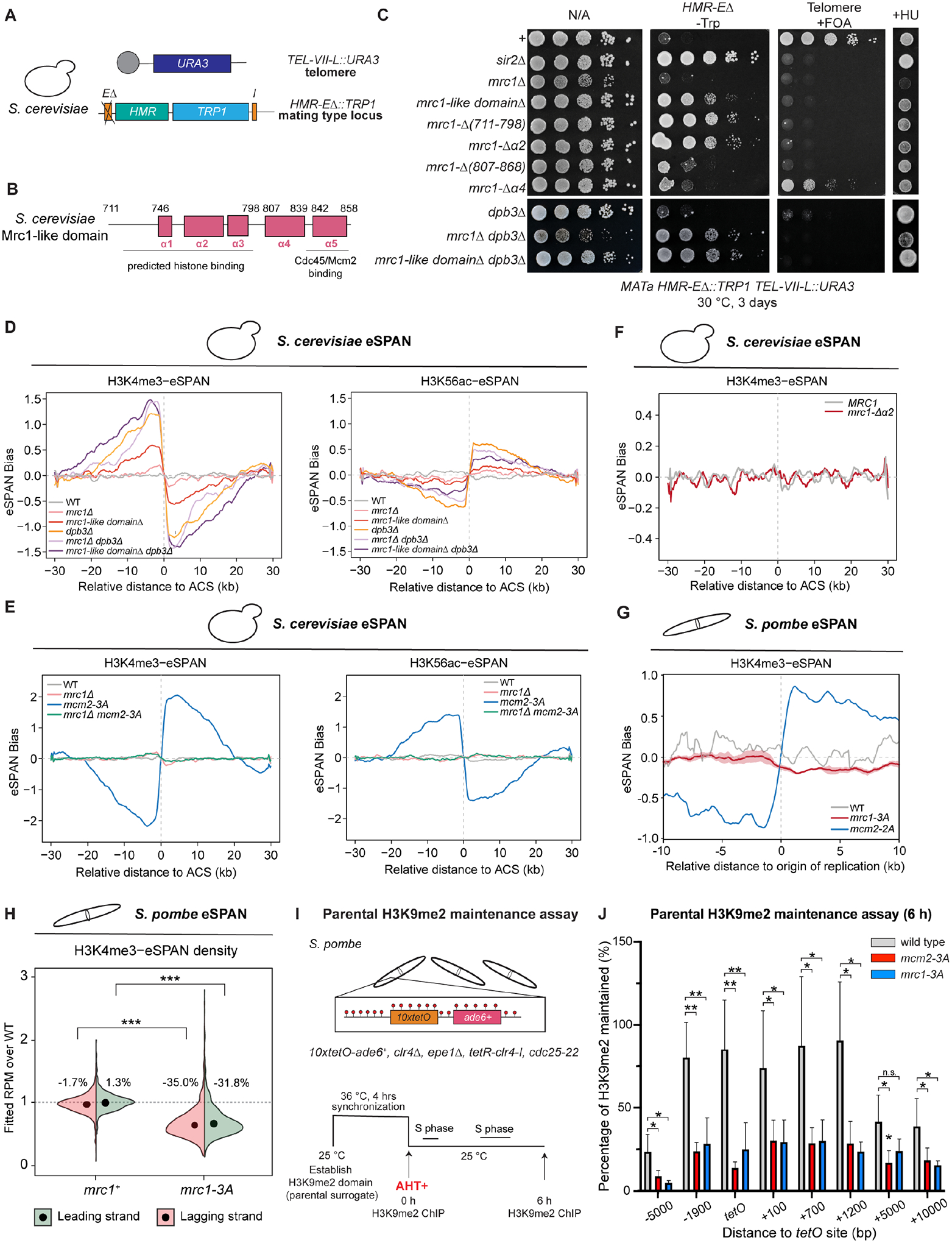
The histone binding domain of Mrc1 promotes parental histone transfer without affecting transfer symmetry. A) Diagram illustrating the dual gene silencing reporter systems in *S. cerevisiae*. B) Diagram of the predicted histone binding domain and Mcm2/Cdc45 interaction region, PDB: 8B9C^104^ and AlphaFold prediction (more details are presented in Figure S7J–P), in the Mrc1-like domain of *S. cerevisiae* Mrc1. C) Growth assays showing the effects of the indicated mutations on silencing and replication stress. D) eSPAN bias of the parental histone surrogate H3K4me3 (left panel) and the new histone surrogate H3K56ac (right panel) distribution around 139 early replicating origins (ACSs) in wild-type (WT*), mrc1*Δ, *mrc1-like domainΔ, dpb3*Δ, *dpb3Δ mrc1Δ*, and *dpb3Δ mrc1-like domainΔ S. cerevisiae* cells. E) eSPAN bias of parental histones surrogate H3K4me3 (left panel) and the new histone surrogate H3K56ac (right panel) around 139 early ACSs in wild-type (WT), *mrc1*Δ, *mcm2-3A*, and *mrc1*Δ *mcm2-3A S. cerevisiae* cells. F) eSPAN bias of the parental histone H3K4me3 distribution in *MRC1, mrc1-α2Δ S. cerevisiae* cells. G) eSPAN bias of parental histone surrogate H3K4me3 distribution around 162 origin of replication in wild-type (WT), *mrc1-3A, mcm2-2A S. pombe* cells. The shading of the bias line plot is the 95% confidence interval of mean value of at least two biological replicates, which is mean ± 2 folds of the standard error. H) Violin plot showing the average of two biological replicates of *S. pombe* eSPAN H3K4me3 density on the leading and lagging strand around the replication origin (2.5 kb upstream of replication origin to 2.5 kb downstream of replication origin). The numbers in the figure represent changes of eSPAN density over wild type cells for each strand. *** indicates p-value < 0.001 (two-sample t-test). I) Diagram illustrating a parental H3K9me2 maintenance assay. Top, diagram of the *S. pombe* reporter system that lacks read-write activity. Bottom, diagram of the designed assay to analyze the maintenance of H3K9me2 in a synchronized cell population after 6 hours after release from cell cycle arrest. J) ChIP-qPCR of parental H3K9me2 in wild-type (WT), *mcm2-2A, mrc1-3A* cells 6 hours after release from cell cycle arrest. A two-tailed two-sample t-test with unequal variance was used for statistical significant test between wild-type and mutant samples. N=5. *, p-value < 0.05, **, p-value < 0.01, n.s., not significant (p=0.068).

We next tested the possible role of the histone binding domain of Mrc1 (Mrc1-HBD) in gene silencing in *S. cerevisiae*, which diverged from *S. pombe* approximately 420 to 330 million years ago. We examined the effect of *mrc1* deletion and mutations on silencing in a sensitized dual reporter *S. cerevisiae* strain, in which the *TRP1* gene is located at the silent mating type *HMR* locus where the *E* silencer is deleted, and the *URA3* gene is located near the left telomere of chromosome VII (Figure 5A–B)^95^. Silencing of the *TRP1* reporter inhibits growth on medium lacking tryptophan (Trp−) whereas silencing of the *URA3* reporter allows cells to grow on medium containing 5-Fluoroorotic acid (FOA+), which is toxic to *URA3*-expressing cells. In the absence of the *E* silencer, establishment of silencing by the *I* silencer is less efficient and silencing may become more sensitive to the loss of parental histone transfer. Establishment of silencing at *TEL-VII::URA3* locus is also less robust than silencing at other telomeres due to the engineered deletion of subtelomeric X’ and Y’ elements^96^. While this reporter system does not separate establishment and maintenance phases of silencing, it provides a sensitive assay for testing the possible effects of specific mutations on a chromatin-dependent silencing mechanism.

As shown in Figure 5C, *mrc1*Δ and mutations in the conserved Mrc1-like domain (*mrc1-*Δ*711–850*, *mrc1-*Δ*711–798*), or in the Mrc1-HBD *(mrc1-*Δα*2*, amino acids 760 to 790) cells were defective for silencing of the telomeric reporter gene *URA3* to nearly the same extent as *sir2*Δ cells in which heterochromatin is not established. The *HMR-E*Δ*::TRP1* locus was fully derepressed in Mrc1-HBD mutant cells but not in *mrc1*Δ cells. It has previously been shown that *mrc1*Δ cells have slightly shortened telomeres^97^, which is known to result in defective telomeric silencing but stronger silencing at the mating type locus^98^. Therefore, the robust silencing observed at the *HMR-E*Δ reporter in *mrc1*Δ cells may result from redistribution of limiting silencing proteins to the *HMR-E*Δ locus, allowing more efficient *I* silencer-dependent establishment, masking the *mrc1*Δ maintenance defect. Deletion of *DPB3* (*dpb3*Δ), which has an established role in parental histone transfer to the leading strand^16^, also had no effect on silencing of the *HMR-E*Δ*::TRP1* locus, but silencing at this locus was lost in *mrc1*Δ *dpb3*Δ double mutant cells (Figure 5C). This suggests that at the *S. cerevisiae HMR-E*Δ*::TRP1* locus Mrc1 and Dpb3 may play redundant roles in the leading strand histone transfer pathway. We conclude that the histone binding domain of Mrc1 plays an evolutionarily conserved role in maintaining silent chromatin domains.

### Mrc1 is required for parental histone maintenance following DNA replication

To test whether the histone binding activity in Mrc1 contributes to the symmetric inheritance of parental histones, we conducted enrichment and sequencing of protein-associated nascent DNA (eSPAN) using histone modifications H3K4me3 and H3K56ac as surrogates for parental and new histones, respectively, in *S. cerevisiae* and *S. pombe* cells (Figure S5A). As expected^16^, in wild-type *S. cerevisiae* cells, we observed no apparent bias of parental and new histone inheritance at daughter strands around 139 early replication origin regions, indicating symmetrical distribution of parental histones at both strands (Figure 5D, Figure S5B–C,H). By contrast, *mrc1*Δ and *mrc1-like domain*Δ (*mrc1-*Δ*711–850*) cells displayed weak preferential transfer of parental histones (H3K4me3) towards the lagging strand (Figure 5D, Figure S5B–C,H). As controls, *dpb3*Δ cells had a strong eSPAN H3K4me3 bias toward the lagging strand, which was enhanced in *dpb3*Δ, *mrc1*Δ and *dpb3, mrc1-like domain*Δ double mutant cells (Figure 5D). New histones (H3K56ac), on the other hand, showed a slight bias towards the leading strands in the mutant cells, suggesting that defects in the transfer of parental histones to the leading strand was partially compensated by new histone deposition (Figure 5D, Figure S5D,I). Consistent with an important role for Mrc1 in governing symmetrical parental histone transfer, the strong leading strand bias of *mcm2-3A* cells^14,15^ was completely reversed in *mcm2-3A*, *mrc1*Δ double mutant cells (Figure 5E). Loss of the entire Mrc1 protein may therefore lead to inefficient recycling of parental histones and suppresses the biased H3K4me3 eSPAN ratios.

Because Mrc1 makes extensive contacts with other replisome components^99–101^ and Mrc1-like domain contains regions that do not directly interact with histones, deletion of the entire Mrc1 or Mrc1-like domain may impact parental histone transfer ratios independently of the histone binding activity of Mrc1. To specifically test whether Mrc1-HBD has intrinsic histone transfer bias, we performed eSPAN experiments using *mrc1* mutations that abolish histone binding without affecting interactions with the replisome: *mrc1-*Δα*2* in *S. cerevisiae* and *mrc1-3A* in *S. pombe*. In support of a specific effect on histone binding, mass spectrometry analysis of Sld5-TAP immunoprecipitations from *mrc1*^+^ and *mrc1-3A S. pombe* cells showed that Mrc1-3A remained associated with the replisome and did not affect the association of other replisome proteins with Sld5 (Figure S6A). Surprisingly, unlike *mrc1*Δ or *mrc1-like domain*Δ, *mrc1-*Δα*2 S. cerevisiae* cells had no apparent strand bias patterns for H3K4me3 or H3K56ac (Figure 5F; Figure S5E–I). Consistent with the *S. cerevisiae* results, eSPAN analysis in *mrc1-3A S. pombe* cells showed no apparent strand bias for H3K4me3, while control *mcm2-2A* cells showed a strong expected leading strand eSPAN bias (Figure 5G; Figure S5J). Therefore, eSPAN analysis of Mrc1 histone binding mutant cells in both *S. cerevisiae* and *S. pombe* suggests that mutations in Mrc1-HBD do not affect symmetrical histone transfer.

Based on the above results, we hypothesize that loss of heterochromatin maintenance in Mrc1 histone binding mutant cells results from reduced parental histone transfer to both daughter DNA strands. Consistent with this hypothesis, eSPAN experiments in *S. pombe* indicated the H3K4me3 density around the origins of replication at the leading and lagging strands are both significantly reduced in *mrc1-3A* cells (lagging strand reduced 35.0%, and leading strand reduced 31.8%, p-value < 0.001) (Figure 5H). We further used ChIP to examine the maintenance of H3K9me2 at the *10XtetO-ade6*^+^ locus in cells that carried a TetR-Clr4-*ΔCD* to establish H3K9me2 at *10XtetO* locus, lacked endogenous Clr4 methyltransferase, and carried a *cdc25–22* temperature-sensitive allele allowing cell cycle arrest at late G2 phase at 36°C and release of synchronized cells from the arrest at 25°C (*tetR-clr4-*Δ*CD*, *clr4*Δ, *cdc25–22*, Figure 5H). *tetR-clr4-*Δ*CD*, *clr4*Δ cells are read-write deficient, allowing us to establish H3K9me2 in cell cycle synchronized cells and then track its recycling following the release of TetR-Clr4-ΔCD and progression through S phase (Figure 5I). Both *mrc1-3A* and *mcm2-3A* cells maintained less H3K9me2 six hours after the release of TetR-Clr4-ΔCD and progression through the cell cycle, indicating that they were defective in recycling parental histones (Figure 5J). These results suggest that Mrc1-HBD distributes histones to both the leading and lagging strand transfer pathways without affecting symmetrical parental histone transfer.

### Distribution of FACT binding sites on the replisome

Since Mrc1 associates with the FACT complex independently of the replisome (Figure 1G)^102^, and previous works showed that the N terminus of Mcm2 binds to histones together with FACT^32^, we hypothesize that Mrc1 and other histone binding proteins in the replisome can co-chaperone histones with FACT. To gain additional insight into the interactions of FACT with the replisome, we performed pairwise AlphaFold-Multimer predictions between FACT subunits and replisome components (Figure S6B). Consistent with the IP-MS results, AlphaFold-Multimer predicted two FACT binding domains (FBD) in Mrc1, which we confirmed by GST-pulldown assays (Figure S6C–D). Mrc1-FBD1 (amino acids 134 to 168) interacts with the Spt16 middle domain (MD) (amino acids 664 to 930) (Figure S6E–G). Mrc1-FBD2 (amino acids 513 to 540) is located near the Mrc1-HBD (amino acids 708 to 809) and interacts with the Spt16 N-terminal domain (NTD) (amino acids 2 to 437) (Figure S6H–J). AlphaFold-Multimer structural predictions show that the Mrc1-HBD may engage an H3-H4 tetramer bound to the Spt16 middle domain (MD), supporting the idea that Mrc1, like Mcm2, may co-chaperone histones together with FACT (Figure S6F,I). However, deletions of Mrc1-FBDs had no effect on heterochromatin maintenance, suggesting that other FACT binding sites on the replisome may compensate for loss of contacts with Mrc1 in vivo.

**Figure 6.**
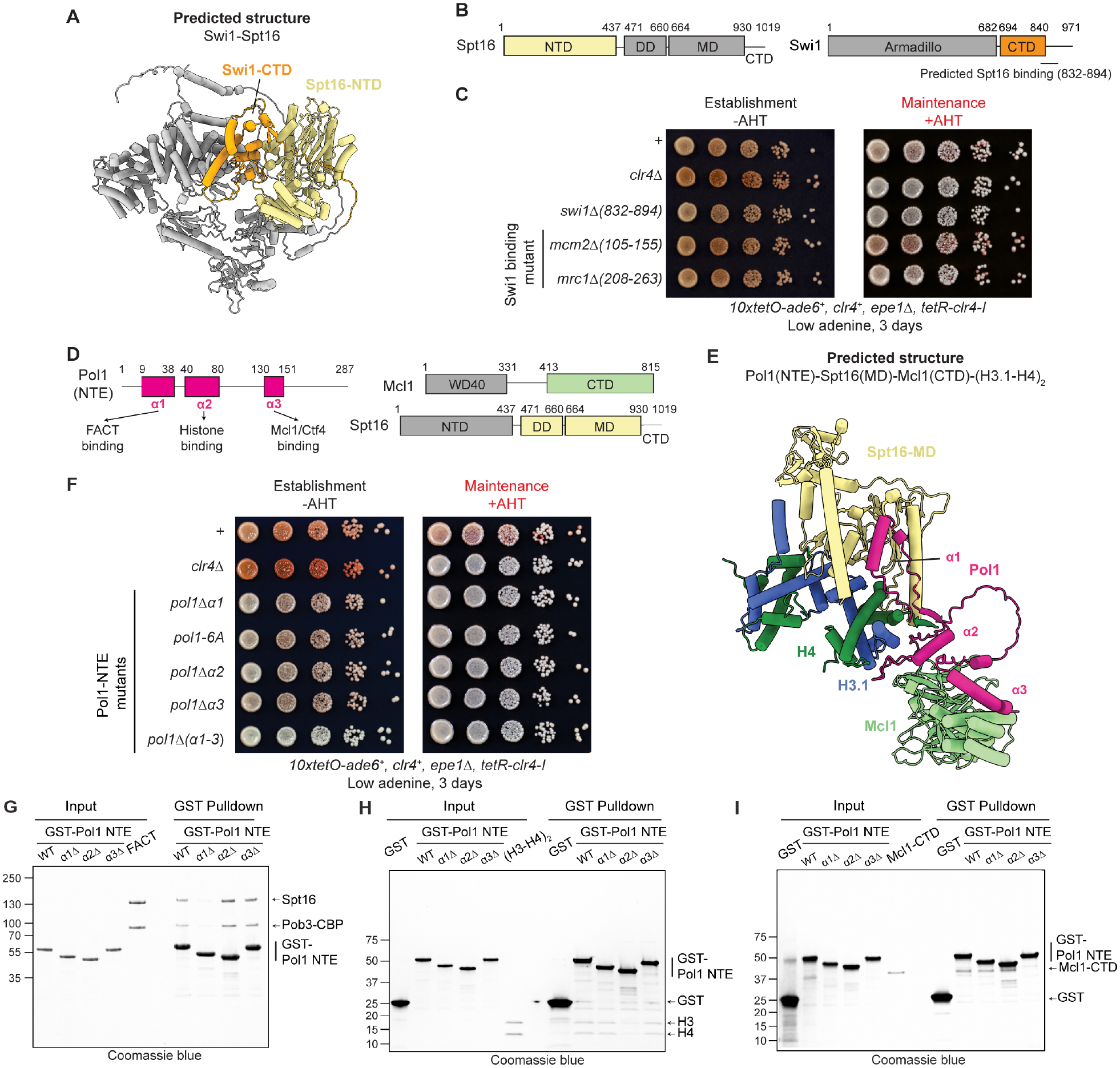
Identification of FACT binding sites on the replisome required for heterochromatin maintenance. A) Predicted structure of Swi1 and FACT subunit Spt16. B) The predicted interacting domains of Spt16 and Swi1 in A are highlighted in yellow and orange, respectively. C) Heterochromatin maintenance assay showing the effects of *swi1*, *mrc1, mcm2* mutations. D) Diagram of regions in the N-terminal extension (NTE) of Pol1 predicted to interact with Spt16, (H3.1-H4)_2_, and the Mcl1 C-terminal domain (CTD). The predicted interacting domains of Spt16 and Mcl1 in F are highlighted in green and yellow, respectively. E) Predicted structure of Pol1-NTE (α1, α2, and α3) with Spt16-middle domain (MD), (H3.1-H4)_2_ and Mcl1-CTD). F) Heterochromatin maintenance assay showing the effect of the indicated *pol1* mutations. G-I) In vitro GST pull down assays showing the interaction of the indicated GST-Pol1-NTE proteins with purified FACT complex (G), (H3-H4)_2_ (H), and Mcl1-(CTD)(I).

AlphaFold-Multimer also identified potential interaction interfaces between Spt16 and the Swi1 subunit of the fork protection complex and the Pol1 subunit of DNA polymerase α (Figure 6A–E; Figure S6B). The predicted Swi1-Spt16 interaction is mediated by the C-terminal domain of Swi1 (Swi1-CTD) and the Spt16-NTD (Figure 6A–B; Figure S7A), which is conserved in *S. cerevisiae* and is one of the previously reported Swi1 domains shown to interact with FACT in pulldown experiments^61^. Deletion of Swi1-CTD (*swi1*-Δ*832–894*) abolished heterochromatin maintenance, suggesting that FACT recruitment via Swi1 may play a role in parental histone transfer (Figure 6C).

In addition to Swi1-FACT, AlphaFold-Multimer predicted Swi1-Mcm2 and Swi1-Mrc1 interactions (Figure S7B–E). The predicted Swi1-Mrc1 interaction interface was previously visualized in a cryo-EM structure of the human replisome^99^ and the Swi1-Mcm2 interface seems to correspond to unassigned density in the human replisome structure^99^ (Figure S7B–E). However, the deletions of these interaction interfaces in Mcm2 and Mrc1 (*mcm2-*Δ*105–155* and *mrc1-*Δ*208–263*, respectively) had no effect on heterochromatin maintenance (Figure 6C), suggesting that they are not required for parental histone inheritance.

The N-terminal extension (NTE) of Pol1, which is predicted to interact with Spt16, is next to the previously identified Pol1-histone binding domain and the Mcl1/Ctf4/WDHD1 binding site (Figure 6D–E; Figure S7F–I)^103^, suggesting that Pol1-NTE is docked on Mcl1 to facilitate parental histone maintenance. Indeed, AlphaFold predicted three adjacent α helices in the N terminus of Pol1 that can simultaneously interact with Spt16-MD (α1, amino acids 9 to 38), (H3-H4)_2_ (α2, amino acids 40 to 80), and Mcl1-CTD (α3, amino acids 130 to 151) (Figure 6D–E; Figure S7F–I). Deletion of any of the 3 α helices or point mutations within α2, previously shown to abolish histone binding^24,35^, abolished heterochromatin maintenance (Figure 6F), highlighting the importance of this Pol1 region. Interestingly, deletion of the entire region encompassing α1-α3 (pol1-Δα1–3) resulted in loss of heterochromatin establishment (Figure 6F). The basis of this establishment defect is presently unclear but suggests a possible role for Pol1 in chromatin assembly beyond histone recycling. Consistent with AlphaFold predictions, in vitro GST pulldown assays indicated that Pol1-NTE encompassing α1-α3 pulled down FACT, H3-H4, and Mcl1. Furthermore, deletion of α1, predicted to interact with FACT, specifically abolished FACT binding (Figure 6G), deletion of α2, predicted to interact with H3-H4, specifically abolished H3-H4 binding (Figure 6H)^24^, and deletion of α3, predicted to interact with Mcl1, specifically abolished Mcl1 binding (Figure 6I)^103^. Together, our data suggests that Pol1, docked on Mcl1, may co-chaperone parental histones together with FACT to promote their transfer to the lagging strand.

## DISCUSSION

**Figure 7.**
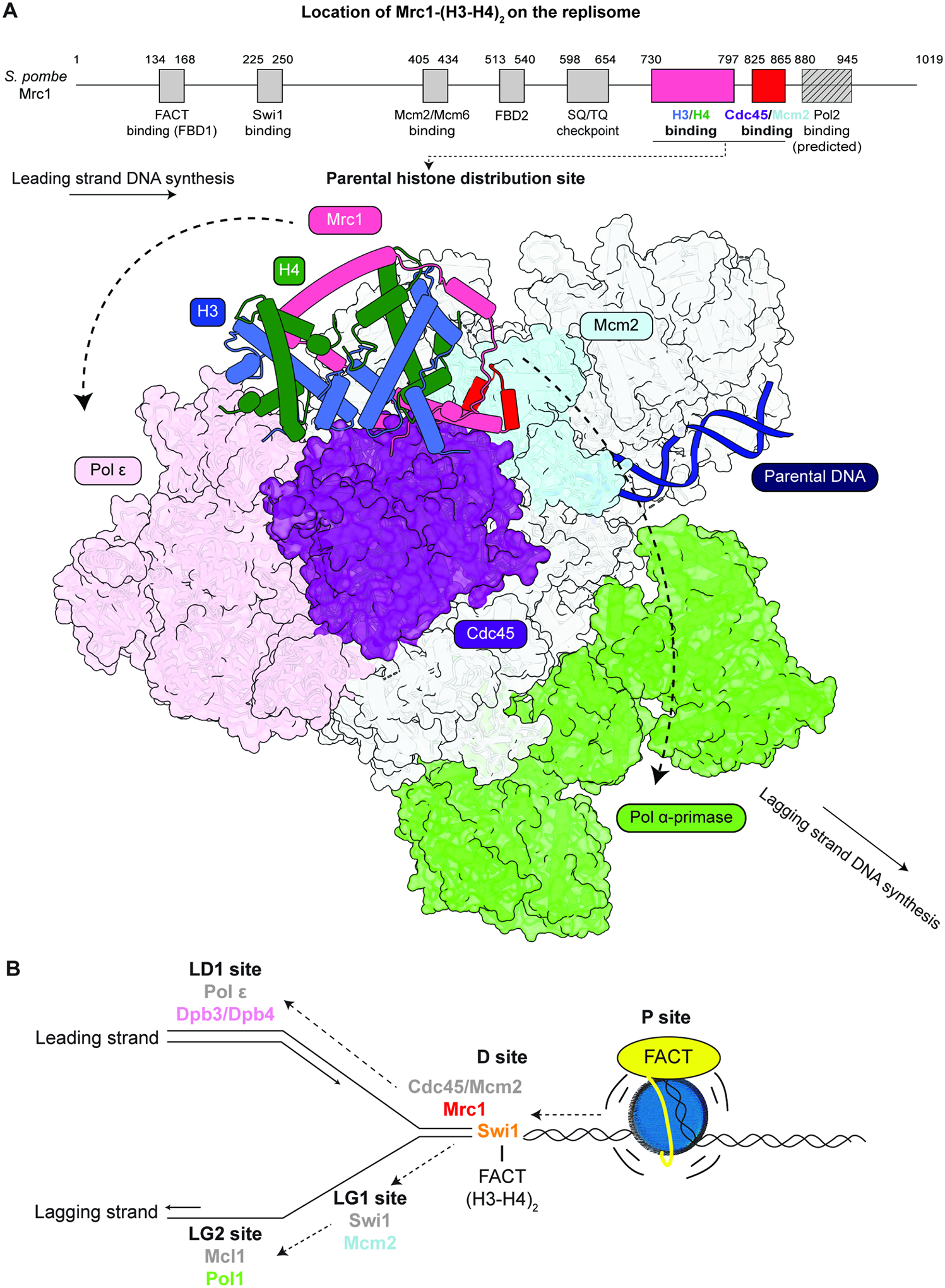
Mrc1 acts as a parental histone distribution site. A) The predicted location of Mrc1-(H3-H4)_2_ on the cryo-EM structure of the replisome (PDB: 8B9C and 7QHS). Top, diagram showing indicated regions in the Mrc1 involved in interaction with multiple replisome components, replication checkpoint signaling, and interaction with histones. The predicted Pol2 interacting region was identified by AlphaFold-Multimer and is consistent with previous biochemical results^101^. The newly identified histone binding region is highlighted in pink and the Cdc45/Mcm2(NTD) interacting region is highlighted in red. Bottom, the predicted structure of Mrc1-like domain/(H3-H4)_2_/Cdc45/Mcm2(NTD) was aligned to the cryo-EM structure (PDB: 8B9C) via the Mrc1-like domain α5 helix. See Figure S7K–P for alignment details. B) Model for DNA replication-coupled directional parental histone transfer with FACT acting as a mobile chaperone. P, Parental site; D, Distribution site; LD1, leading strand site 1; LG1 and LG2, lagging strand sites. See text for details.

In this study, we identify the Mrc1/CLASPIN subunit of the fork protection complex as an H3-H4 tetramer chaperone critical for parental histone maintenance during DNA replication and heterochromatin inheritance. Our findings suggest that Mrc1/CLASPIN together with FACT and other replisome components form a network of chaperones that coordinate the transfer of intact parental histone H3-H4 tetramers to newly replicated DNA. The location of the Mrc1 histone binding domain and the fork protection complex on the replisome and the requirement for Mrc1 in parental histone transfer to both daughter DNA strands suggest that Mrc1-HBD acts as part of a distribution center for the initial capture and transfer of histones to the leading and lagging strand pathways (Figure 7; Figure S7J–P).

Our findings suggest broad roles for Mrc1 and Mrc1-HBD in parental histone transfer to newly replicated DNA. eSPAN analysis of cells carrying a full deletion of *mrc1*^+^ (*mrc1*Δ) or deletions of the Mrc1-like domains extending beyond its histone binding domain display a weak bias for parental histone transfer to the lagging strand in *S. cerevisiae*, suggesting that symmetrical histone transfer requires Mrc1. Larger Mrc1 deletions furthermore greatly enhance the lagging strand bias of *dpb3Δ* cells suggesting that Mrc1 and Dpb3 function together in the leading strand transfer pathway. However, *S. cerevisiae* cells with a deletion of the Mrc1-α2, which specifically disrupts H3-H4 binding, do not affect the eSPAN bias ratios. Similarly, *S. pombe* Mrc1 mutations that specifically disrupt H3-H4 binding (*mrc1-3A*) do not affect eSPAN bias ratios but greatly reduce the maintenance of parental H3K9me after DNA replication. These observations suggest distinct roles for the Mrc1-HBD and other Mrc1 domains in parental histone transfer that include roles for Mrc1 in coordinating the activities of other replisome components to ensure symmetrical parental histone transfer (via domains outside its HBD) (Toda et al., 2024) and direct distribution of parental histone to both the leading and lagging strand pathways (via its HBD).

The available cryo-EM structures of the replisome^104,105^ and AlphaFold structural predictions suggest that Mrc1 makes extensive interactions with other replisome components and allow us to pinpoint the location of Mrc1-HBD (Figure 7A; Figure S7J–P). The interactions of Mrc1 regions adjacent to its HBD with the Cdc45/Mcm2 components of the replicative helicase suggest that the Mrc1-HBD is located at a central position on the replisome from which it may act as a distribution site for the transfer of parental H3-H4 tetramers to either the leading or the lagging strands (Figure 7; Figure S7J–P). Beyond its HBD, Mrc1 interacts with multiple components of the replisome, including other subunits of the fork protection complex, Cdc45, Mcm2, and the catalytic subunit of DNA polymerase ε^99–101,104^ (Figure 7A, Table S6). The complete absence of Mrc1 may therefore alter the structure of the replisome in a way that globally disrupts strand-specific parental histone transfer. In this model, Mrc1 would act as a key modulator of the overall replisome conformation ensuring that multiple histone binding domains are properly orientated to achieve symmetrical parental histone transfer. This model also provides an explanation for distinct phenotypes of mutations in the Mrc1-HBD compared to deletion of the entire Mrc1 or mutations outside its HBD. It also raises the exciting possibility that regulation of Mrc1 interactions may contribute to biased parental histone transfer at specialized replication forks or cells^106,107^.

Compared to other histone-binding replisome components, Mrc1 contains a binding interface with the entire H3-H4 tetramer through physical properties that resemble nucleosomal components that bind to the H3-H4 tetramer in the nucleosome core particle. This mode of H3-H4 binding may be critical for the transfer of intact H3-H4 tetramers to newly synthesized DNA. By contrast, experimental^33,34^ and predicted structures suggest that Mcm2, Pol1, and Dpb3/Dpb4 only bind to H3-H4 dimers (Figure S3A–E) and are therefore likely to have a more stringent requirement for the FACT complex in transporting H3-H4 tetramers. Several recent studies show that FACT favors binding to destabilized over intact nucleosome substrates^47,48^ and is required for chromatin replication in vitro^52^. Parental nucleosome disassembly in response to the force exerted by the replicative CMG helicase may be facilitated by binding of FACT to the partially disassembled nucleosome (Figure 7B), similar to the association of FACT with partially disrupted nucleosomes during transcription elongation^49,50^. In addition, FACT has domains that interacts with the catalytic subunit of DNA polymerase α Pol1^58^, RPA^37,60^, Mcm2-7 complex^32,108^, Tof1/Swi1^61^, and Mrc1 (this study). The requirement for the FACT binding sites on Swi1 and Pol1 in epigenetic maintenance of heterochromatin support the idea that FACT-replisome interactions contribute to parental histone recycling.

Our analysis of the locations of histone binding domains on the structure of the replisome^99,100,104,105^ allows us to propose stepwise pathways for the transfer of parental histones to newly replicated DNA (Figure 7B). We propose that the parental nucleosome is destabilized by the CMG helicase leading to recruitment of the FACT complex and further nucleosome disassembly^46,48^ (Figure 7B, Parental or P site). FACT captures parental histones from the P site and is then recruited to the replisome through its interaction with the Swi1 subunit of the fork protection complex (Figure 7B). Since Swi1 interacts with Mrc1, Mcm2, FACT^61^, and histones, and Mrc1 contributes to parental histone transfer to both daughter DNA strands, we propose that Swi1 and Mrc1-HBD forms a distribution hub (D site) for transfer of the FACT-H3-H4 complex to the leading or lagging strands (Figure 7B). Leaving the D site, the FACT-histone complex may be captured by Dpb3-Dpb4 (Leading site 1, LD1 site) for deposition onto the newly synthesized leading DNA strand. For the lagging strand pathway, the FACT-H3-H4 complex would be transferred from the D site to the Mcm2 histone binding domain (Lagging site 1, LG1 site) and to Pol1 (LG2 site) for deposition of histones onto the lagging strand (Figure 7B). The transfer mechanism is dynamic and may rely on intermediate states in which Mrc1-HBD directly hands off (H3-H4)_2_ to other histone binding proteins in the replisome along the leading or lagging strand pathways. This idea is supported by the apparent extended and the likely flexible structure of Mrc1 and AlphaFold predictions suggesting that Mrc1 and each of the histone binding proteins along the leading and lagging strands can simultaneously associate with (H3-H4)_2_ (Figure S8).

### Limitations of the Study

The complexity of the replisome, together with the large distances that parental histone must travel from the front of the replisome to newly replicated DNA, suggest that our understanding of the transfer pathway is still rudimentary. Future experiments are required to understand how the Swi1-Mrc1 hub coordinates the symmetrical and directional transfer of parental histones to the leading and lagging strand binding sites before their deposition on newly synthesized DNA. The proposed order of the binding and transfer events, as well as the AlphaFold predicted structures of intermediate parental histone transfer states, also require further experimental demonstration.

## STAR★METHODS

### RESOURCE AVAILABILITY

#### Lead contact

Further information and requests for reagents or resources should be directed to and will be fulfilled by the lead contact, Danesh Moazed (danesh@hms.harvard.edu). The materials generated in this study will be provided without restriction.

#### Materials availability

Resources and materials generated in this study are available upon request and the request should be directed to lead contact Danesh Moazed.

#### Data and code availability

- The raw gel, membrane, silencing assay images were deposited at Mendeley Data at doi: 10.17632/jhzmfr8bbs.1 and are publicly available on the date of publication. All AlphaFold-Multimer-predicted structures and modeled structures are deposited on ModelArchive under the accession number ma-dm-hisrep and are publicly available on the date of publication. *S. cerevisiae* eSPAN data are deposited at Genome Research Archive (accession number CRA011810) and *S. pombe* eSPAN *mrc1-3A* data are deposited at Genome Expression Omnibus (269383) and are publicly available on the date of publication. Accession numbers for all datasets are listed in the key resource table.
- The codes used to generate and analyze the datasets were deposited at Mendeley Data at doi: 10.17632/jhzmfr8bbs.1 and are publicly available on the date of publication.
- Any additional information that is required for reanalyzing the data reported in this study is available from the lead contact upon request.

### EXPERIMENTAL MODEL AND STUDY PARTICIPANT DETAILS

#### Plasmids

All plasmids used in this study were generated using Gibson Assembly^109^, except for CRISPR-based genome editing plasmids used for construction of some of the *S. pombe* mutant cells, which were generated using Golden Gate ligation^110^. Antibiotics resistant gene-containing plasmids pFA6a-*kanMX6*, *natMX6*, *hphMX6*, *bsdMX* were used as the backbones to generate plasmids to amplify PCR fragments for yeast transformation. pGEX-6p-1 containing GST followed by the 3C protease cleavage site was used as the backbone to generate GST-fusion protein constructs for recombinant protein expression and purification.

#### Yeast strains

All *S. pombe* and *S. cerevisiae* strains were generated using homologous recombination-based mutagenesis with PCR amplified fragments that carried homology arms and desired mutations^111,112^ except for *swi3*Δ, *rfa3*Δ and *ctf18*Δ *S. pombe* strains, which were generated using CRISPR-Cas9^110^. All *S. pombe* and *S. cerevisiae* strains used in this study are listed in Table S1, respectively. gRNAs used to delete *swi3*^+^*, rfa3*^+^*, ctf18*^+^ are listed in Table S1.

### METHOD DETAILS

#### Yeast reporter assays

For heterochromatin maintenance and replication stress assays, *S. pombe* cells were cultured in YES media overnight and then diluted to 1.0×10^5^ cells/mL (OD_600_=1.0, Nanodrop). Cells were washed with sterile water and resuspended to 4×10^5^ cells/mL (OD_600_=4.0, Nanodrop). Serial dilutions (1, 1:10, 1:100, 1:1000, 1:10000) of cells were then spotted on YE (low adenine), YE+10 μM anhydrotetracycline (AHT, Cayman chemical), or YES+5 mM hydroxyurea (Sigma-Aldrich) plates to assay heterochromatin establishment, maintenance, and replication stress, respectively. The plates were photographed after incubation at 32 °C for 3 days. For DNA-sequence dependent heterochromatin maintenance assays, *S. pombe* cells were prepared as above and plated on YES, EMMc-Ura (EMM powder, Sunrise Science Products), or EMMc+FOA (5-FOA, Goldbio) plates to assay heterochromatin establishment and maintenance at the mating type locus. To quantify the percentage of silent colonies in the heterochromatin maintenance assay, 60 μL 1:1000 dilute cells from the density of OD_600_=1 were plated on YE+AHT plates. For heterochromatin spreading assay, *S. pombe* cells were prepared as above and plated on YE plate. For *S. cerevisiae* gene silencing assay, cells were cultured in YEPD+Ade+Trp medium overnight and diluted to OD_600_=1.0 (Nanodrop). Cells were washed with water and resuspend to 4×10^5^ cells/mL (OD_600_=4.0, Nanodrop). Serial dilutions (1, 1:10, 1:100, 1:1000, 1:10000) of cells were then spotted on YEPD+Ade+Trp, SC-Trp, SC+FOA, or YEPD+Ade+Trp+50 mM HU plates to assay cell growth, reporter gene silencing at the mating type locus and telomere, and replication stress phenotype, respectively. The plates were photographed after incubation at 30 °C for 2 days. Images were captured by Nikon D70 under the control of Nikon Camera Control Pro. Global adjustment of contrast and saturation of the images were conducted by Adobe Lightroom for the presentation.

#### Chromatin immunoprecipitation

To prepare ChIP samples, *S. pombe* cells were cultured in YES medium overnight and diluted to OD_600_ = 0.2 in YES medium and processed for ChIP as previously described^113^ with modifications. For heterochromatin maintenance phase experiment, *S. pombe* cells were cultured with 10 μM AHT for 24 hours. For cell cycle synchronization experiment, *cdc25–22 S. pombe* cells were first cultured at 25 °C in mid log-phase, then transferred to 36 °C culture for 4 hours to arrest at late G2 phase, and then immediately cool down in water bath at 25 °C supplemented with 10 μM AHT and cultured for another 6 hours at 25 °C to release from late G2 phase and resume cell cycle. After reaching OD_600_=2~3, cells were crosslinked in 1% methanol-free formaldehyde (16% w/v formaldehyde, ThermoFisher) for 15 min at room temperature, followed by quenching using 100 mM glycine for 5 min at room temperature. Cells were then pelleted by centrifuging at 5,000 rpm for 1 min at 4 °C, washed with 1 mL cold TBS (20 mM Tris, 150 mM NaCl) buffer, flash frozen in liquid nitrogen, and stored at −80 °C. Frozen cell pellets were resuspended in ChIP lysis buffer (50 mM HEPES-KOH, pH 7.5, 140 mM NaCl, 1% Triton X-100, 0.1% SDS, 0.1% Na-deoxycholate, 1 mM EDTA, 1 mM PMSF supplemented with cOmplete protease inhibitor cocktail(Sigma-Aldrich)). 1 mL acid-wash glass beads were added and cells were lysed with MagNA Lyser (Roche) using the program: 3 rounds of 90 s with 4,500 rpm and 1 round of 45 s with 5,000 rpm. Cells were placed in ice-water slush for 1 min to cool down in between each cycle. The lysate was then resuspended to 1 mL and sonicated in millTUBE 1 mL AFA fiber (Covaris) on Covaris E220 evolution sonicator at 4 °C using the program: 5% duty cycle, 140 PIP, 200 cycle per burst for 12 min. The lysate was then centrifuged at 13,200 rpm for 15 min at 4 °C. The supernatant was collected, 5% of which is saved as input. The remainder of each sample was incubated with Dynabeads protein A (Invitrogen) conjugated anti-H3K9me2 antibody (Abcam) at 4 °C for 3 hours. 30 μL protein A magnetic beads were incubated with 2 μg anti-H3K9me2 antibody at 4 °C for 1 hour and then added to each sample. After incubation, magnetic beads were collected using a magnetic stand and washed with ChIP lysis buffer three times and with prechilled TE once. Magnetic beads were then eluted with 100 μL ChIP elution buffer A (50 mM Tris-HCl, pH 8.0, 10 mM EDTA, 1% SDS) and 150 μL ChIP elution buffer B (TE with 0.67% SDS) for 5 min at 65 °C with 1,400 rpm on an Eppendorf Thermomixer F1.5. Eluted fractions were combined and incubated at 65 °C overnight to reverse crosslinks. Samples were then treated with ChIP protein digestion buffer containing 3 μg proteinase K (Roche), 100 mM LiCl, and 5 μg glycogen (Roche) in TE at 55 °C for 1 hour. ChIP and input DNA were then purified using phenol-chloroform extraction followed by ethanol precipitation. Percent of input of ChIP DNA was then analyzed by quantitative PCR of input and ChIP DNA on Applied Biosystems QuantStudio 7 flex. All qPCR primers used for ChIP experiments are listed in Table S2.

#### Immunoprecipitation

Immunoprecipitations of replisome factors were carried as described^32^ with modifications. *S. pombe* cells were cultured overnight at 32 °C in YES medium, diluted to OD_600_=0.05 in YES medium, and incubated in a shaker at 32 °C for 14 hours. 1×10^10^ cells were harvested by centrifuging at 5,000 rpm for 10 min at 4 °C and cell pellets were washed once with 25 mL prechilled TBS buffer. The cell pellets were weighed and resuspended in 1/5 volume of resuspension buffer (20 mM HEPES-KOH pH 7.5, 100 mM KOAc, 5 mM Mg(OAc)_2_, 0.25% Triton X-100, 1 mM EDTA, 10% (v/v) glycerol). Cell resuspensions were then added into liquid nitrogen dropwise to form frozen yeast popcorn. Cells were then broken by grinding the yeast popcorn using Freezer/Mill 6875D with 12 cycles of 90 s vortex, 2 min cool (speed: 10 CPS) and stored in −80 °C. Ground yeast powder was resuspended in lysis buffer (20 mM HEPES-KOH, pH 7.5, 100 mM KOAc, 5 mM Mg(OAc)_2_, 0.25% Triton X-100, 5 mM NaF, 5 mM β-glycerophosphate, 1 mM EDTA, 1 mM PMSF, 1 mM DTT, 10% (v/v) glycerol, supplemented with Roche cOmplete protease inhibitor and protease inhibitor cocktail (Sigma, P8215)), treated with 1000 U/mL Benzonase (Santa Cruz Biotechnology, Catalog No. sc-391121C) for 1 hour at 4 °C. The lysate was centrifuged for 3 min and then 15 min at 13,200 rpm. The supernatant was then incubated with antibodies crosslinked with magnetic beads at 4 °C for 3 hours. Magnetic beads were collected on a magnetic stand, washed with lysis buffer four times, and eluted using 0.5 M NH_4_OH at 37 °C for 20 min. Elutions from beads were then dried in a speed vacuum and analyzed by SDS-PAGE, western blot and mass spectrometry. For TAP immunoprecipitation of Mrc1 or Sld5 proteins, Rabbit IgG (Sigma, 15006) was conjugated to Dynabeads M270 Epoxy (Invitrogen, 14302D) and stored in 1xPBS+0.02% sodium azide at 4 °C before being used for immunoprecipitation. For FLAG immunoprecipitation of Mrc1 proteins, anti-FLAG M2 antibody (Sigma, F1804) was incubated with Dynabeads Protein G (Invitrogen, 10004D) overnight before being used for immunoprecipitation. All antibody-conjugated magnetic beads used in immunoprecipitation were first crosslinked with 14.8 mM dimethyl pimelimidate (DMP, Invitrogen, 21667) in 10 bead-volume of crosslinking buffer (0.2 M sodium borate, pH 9) at room temperature for 30 min, followed by quenching using 10 bead-volume of 0.2 M ethanolamine (Sigma, E9508) at room temperature for 90 min. The spectral counts of proteins identified by mass spectrometry are listed in Table S2–5.

#### Label-free mass spectrometry

Label-free mass spectrometry analysis was performed using on-bead digestion. In solution digestion was performed on beads from immunoprecipitations. 20 μl of 8 M urea (Sigma-Aldrich), 100 mM EPPS (Sigma-Aldrich) pH 8.5 were added to the beads. 5 mM TCEP was added, and the mixture was incubated for 15 min at room temperature. 10 mM of iodoacetamide was then added for 15min at room temperature in the dark. 15 mM DTT was then added to consume any unreacted iodoacetamide. 180μl of 100 mM EPPS pH 8.5 was added to reduce the urea concentration to <1 M, followed by the addition of 1 μg of trypsin (Promega) and incubated at 37 °C for 6 h. The solution was acidified with 2% formic acid and the digested peptides were desalted via StageTip, dried via vacuum centrifugation, and reconstituted in 5% acetonitrile, 5% formic acid for LC-MS/MS processing. Mass spectrometry equipment and parameters used in this study are summarized in Table S3.

#### Taq-based gene-targeted random mutagenesis

Yeast strain SPY9210 (*mrc1-W620STOP-ura4/hphMX6*) was used for the mutagenesis. In brief, cells were transformed with full length Mrc1 fragments generated by Taq polymerase-mediated PCR to replace the missing C terminus of *mrc1*, *ura4-hphMX6* drug cassette to generate a complete *mrc1* allele with random mutations generated by Taq polymerase during PCR. Transformants were selected on FOA plates with two rounds of replica plates. Transformants were then plated on YE, YE+10 μM AHT, YES+5 mM HU and screened for colonies that display red color on YE plates, white color on YE+AHT plates, and viability on YES+HU plates. Candidate colonies were streaked on the YE+AHT plates for single colony purification and candidates with variegated color displayed on the YE+AHT plates were discarded. Cells grown from a single colony from individual candidates were then assayed again on YE, YE+AHT, YE+HU plates with serial dilutions to confirm maintenance-specific defects. The entire *mrc1* gene from each candidate was amplified, followed by Sanger sequencing to identify mutations.

#### Identification and alignment of Mrc1-like domain among eukaryotic species

Mrc1-like domain is annotated among fungi as the PF09444. Additional Mrc1-like domains among other eukaryotic species were identified by aligning fission yeast Mrc1-like domain with full length Mrc1/CLASPIN homologs in each species using Clustal Omega through UniProt with 5 iterations^114^. Mrc1-like domains from each species were used as the input for AlphaFold-Multimer structural predictions to narrow down the Mrc1-histone binding domain used in in vitro biochemical experiments. Multiple sequence alignment of nine Mrc1-histone binding domain among model eukaryotic organisms were performed using Clustal Omega through UniProt with 5 iterations and visualized by JalView^115^. The evolutionary conservation of amino acids threshold was 25.

#### Structural predictions and analysis of protein-protein interactions

All structural predictions of protein-protein interactions were performed using template-free AlphaFold-Multimer v2 and v3 through ColabFold from Chimera X, Google Colab, or localColabFold at Harvard Medical School local computational cluster O2^83–88^. The configurations of each structural prediction are listed in Table S6.

For the evaluation of the protein-protein interactions between a group of predictions from AlphaFold-Multimer v3, interface predicted template modeling (ipTM) scores^87^ of the first rank structure and average ipTM scores of all five structures were collected and visualized with a heatmap generated by a Python3 script with the assistance of ChatGPT (openAI).

For the analysis of the features of predicted Mrc1-like domain-H3-H4 tetramer structure, published crystal structure of nucleosome core particle (PDB: 1AOI)^26^ was used to align with the predicted structures. To identify the location of Mrc1 on the replisome, published cryo-EM replisome structures were used to model and align: i) the predicted interaction between N-terminal Mrc1 and Swi1/TIMELESS with the published cryo-EM human replisome structure (PDB: 7PFO, Figure S7D–E)^99^, ii) the predicted interaction between Mrc1-like domain and Cdc45/Mcm2(NTD) with the published cryo-EM *S. cerevisiae* replisome structure (PDB: 8BC9)^104^. The location of Mrc1-like domain-H3-H4 tetramer was aligned to a modelled replisome structure by aligning two published cryo-EM *S. cerevisiae* replisome structures (PDB: 8BC9 and 7QHS)^104,105^.

For evaluation of the predicted *S. pombe* Mcm2-H3.1-H4 tetramer structure, the predicted structure was aligned to published crystal structure of human MCM2-HBD-H3.3-H4 tetramer (PDB: 5BNV)^33^. For evaluation of predicted *S. pombe* Spt16-H3.1-H4 tetramer structure, the predicted structure was aligned to published crystal structure of human Spt16-MD/AID-H3.1-H4 tetramer (PDB: 4Z2M)^46^.

All structural analysis was performed on UCSF Chimera X (daily build version)^116^. All predicted structures listed in Table S6 are available to download on ModelArchive with the following link: 10.5452/ma-hisrep.

#### Purification of recombinant GST-fused Mrc1-like domain proteins

BL21-CodonPlus competent cells were transformed with pGEX-6p-1 vectors expressing the fusion of GST-tag and fragments of *S. pombe* Mrc1-like domain. BL21-CodonPlus competent cells carrying pGEX-6p-1 vectors were cultured in 1–3 L Terrific Broth (US Biological) media with 100 μg/mL ampicillin and 25 μg/mL chloramphenicol and induced with 2% ethanol and 0.2 mM IPTG at 20 °C for 4 hours with shaking at 220 rpm starting with OD_600_=0.7~0.9. Cells were collected by centrifugation at 7,000 rpm for 20 min at 4 °C. Cell lysate were generated as described above and incubated with 1 mL Glutathione Sepharose 4 Fast Flow resin (Cytiva) at 4 °C for 1 h with rotation. The resin was collected by centrifugation at 4,000 rpm for 5 min at 4 °C and washed with Wash/Equilibrium buffer four times. The resin was then equilibrated in the elution buffer (20 mM HEPES-NaOH, pH 7.5, 100 mM NaCl, 1 mM DTT, 10% (v/v) glycerol, 0.05 mg/mL insulin (Sigma-Aldrich)). 10 μg 3C protease was added into the elution buffer to cleave the Mrc1-like domain from the GST-tag at 4 °C overnight with rotation. Supernatant containing the eluted protein was collected from the resin and subjected to HiTrap Q HP 1 mL (Cytiva). The protein was eluted with a 20-column volume (CV) gradient of NaCl from 100 mM to 1000 mM. *S. pombe* Mrc1-like domain eluted at around 350 mM NaCl. Peak fractions were collected and concentrated using Amicon 10 MWCO Ultra-4 Centrifugal Filter Unit (Sigma-Aldrich). Sample was then injected into Superdex 200 increase 10/300 GL at SEC-M buffer (20 mM HEPES-NaOH, pH 7.5, 350 mM NaCl, 1 mM DTT, 10% (v/v) glycerol). Peak fractions were collected and concentrated again using Amicon 10 MWCO Ultra-0.5 centrifugal Filter Unit (Sigma-Aldrich).

#### In vitro reconstitution of Mrc1-like domain-H3-H4 tetramer complex

Stoichiometric amounts of Mrc1-like domain (stored in 20 mM HEPES-NaOH pH 7.5, 350 mM NaCl, 1 mM DTT, 10% (v/v) glycerol) and reconstituted H3-H4 tetramer complex (stored in 10 mM HEPES-KOH pH 7.5, 1 M NaCl, 0.5 mM DTT, 50% (v/v) glycerol) were mixed on ice and incubated for 10 min. The concentration of Mrc1-like domain was normalized such that the final NaCl concentration in the mixed sample was 500–550 mM. 500 μL of the reconstituted sample was centrifuged at 15,000 rpm at 4 °C for 15 min and injected into Superdex 200 increase 10/300 GL (GE healthcare) at SEC-HM buffer (20 mM HEPES-NaOH, pH 7.5, 500 mM NaCl, 1 mM DTT, 10% (v/v) glycerol). Fractions were collected and analyzed by SDS-PAGE and Coomassie staining.

#### Mass photometry

Mass photometry experiments were performed using Refeyn TwoMP at Harvard Medical School Center for Macromolecular Interactions (CMI) core facility. In brief, 10–20 nM purified Mrc1-like domain, H3-H4 tetramer, or Mrc1-like domain-H3-H4 tetramer complex eluted from the Superdex 200 increase 10/300 GL were added on the slide. Movies were recorded for 30 or 60 seconds. 10 nM-20 nM of mixed BSA (66 kDa) and thyroglobulin (660 kDa) samples were diluted in the MP-assay buffer (20 mM HEPES-KOH, pH 7.5, 500 mM NaCl) right before the measurement to generate a calibration curve. The calibration curve was applied to the samples to estimate the molecular weight of objects recorded in the movies collected by Refeyn AcquireMP. Data were analyzed and visualized in Refeyn DiscoverMP.

#### Size exclusion chromatography with multi-angle light scattering (SEC-MALS)

SEC-MALS experiments were performed with the SEC-MALS system at Harvard Medical School CMI core facility. The SEC-MALS contains an Agilent 1260 Infinity LC System with variable UV detector connected with a Superdex 200 increase 3.2/100 column (Cytiva), a Wyatt Dawn Heleos II MALS detector, and a Wyatt Optilab T-rEX Refractive Index Detector. The SEC column was equilibrated with SEC-MALS buffer (20 mM Tris-HCl, pH 7.5, 500 mM NaCl, 0.5 mM TCEP) overnight at 25 °C. First, 80 μL 30 μM monodispersed BSA (Thermo Scientific) was spun at 14,000 rpm for 10 min and injected into the SEC-MALS system at the flow of 0.045 mL/min at 25 °C through the Agilent autosampler. Peak alignment, band broadening, light scattering detector normalization were performed on the monodispersed BSA monomer peak. Then 80 μL 25–50 μM Mrc1-like domain, H3-H4 tetramer, or Mrc1-like domain-H3-H4 tetramer complex samples were applied to SEC-MALS using the same conditions as the BSA sample. Data were analyzed under the BSA control setting and visualized using ASTRA (version 7.3.2.21).

#### Purification of *S. pombe* Mcl1-CTD domain

BL21-CodonPlus competent cells were transformed with pET28a vectors expressing the fusion of 6xHis-SUMO and *S. pombe* Mcl1-CTD domain. pET28a vector containing BL21-CodonPlus competent cells were cultured in 1 L LB media with 50 μg/mL ampicillin and 25 μg/mL chloramphenicol and induced with 2% ethanol and 0.2 mM IPTG at 20 °C for 4 hours with shaking at 220 rpm starting with OD_600_=0.7~0.9. Cells were collected by centrifugation at 7,000 rpm for 20 min at 4 °C. Cell lysate were generated as described above with the addition of 20 mM imidazole and in the absence of EDTA. Clear lysate was incubated with 1 mL chelating resin at 4 °C for 30 min with rotation. The resin was put on a chromatography column and washed with Wash/Equilibrium buffer (40 mM imidazole) five times. The resin was then equilibrated in the elution buffer (20 mM Tris-HCl, pH 7.5, 500 mM NaCl, 400 mM imidazole, 2 mM β-mercaptoethanol, 10% (v/v) glycerol). Ulp1 protease was added into the elution buffer to cleave the Mcl1-CTD domain from the 6xHis-SUMO in a dialysis buffer (20 mM Tris-HCl, pH 7.5, 100 mM NaCl, 20 mM imidazole, 2 mM β-mercaptoethanol, 10% (v/v) glycerol) at 4 °C overnight. Supernatant containing the eluted protein was subjected to chelating resin once to remove 6xHis-SUMO. Sample was then subjected to HiTrap Q HP 1 mL (Cytiva) with a 20 CV gradient of NaCl from 100 mM to 1 M. Peak fractions containing Mcl1-CTD domain was eluted around 220 mM NaCl and concentrated using Amicon 10 MWCO Ultra-4 Centrifugal Filter Unit. The protein was then further purified Superdex 200 increase 10/300 GL in 20 mM Tris-HCl, pH 7.5, 200 mM NaCl, 1 mM DTT, 10% (v/v) glycerol.

#### Purification of *S. pombe* FACT complex

The FACT complex was purified as described previously^48^ with modifications. Endogenously FACT was purified from Pob3-TAP tagged *S. pombe* strain and overexpressed FACT was purified from *S. pombe* strain overexpressing Spt16, Pob3-TAP driven by *nmt1* promoter in EMMc media. For endogenous FACT purification, Yeast popcorn from 1 L cell culture was prepared as described above for replisome purifications. The yeast popcorn was resuspended in lysis buffer-FE (20 mM HEPES-KOH pH 7.5, 600 mM KOAc, 5 mM Mg(OAc)_2_, 0.01% CHAPS (anatrace), 0.01% octyl-glucoside (anatrace), 1 mM EDTA, 1 mM PMSF, 1 mM DTT, 10% (v/v) glycerol supplemented with Roche cOmplete protease inhibitor). Supernatant was prepared as described above for replisome purifications and incubated with IgG-conjugated Dynabeads or IgG Sepharose 6 Fast Flow affinity resin (Cytiva) at 4 °C for 2 hours with rotation. The magnetic beads or resin were collected and wash with lysis buffer four times. The beads were then equilibrated in elution buffer (20 mM HEPES-KOH pH 7.5, 150 mM KOAc. 5 mM Mg(OAc)_2_, 1 mM EDTA, 1 mM PMSF, 1 mM DTT, 10% (v/v) glycerol). FACT complex was eluted from magnetic beads or resin with TEV protease at room temperature for 1 hour with rotation. For overexpressed FACT purification, yeast popcorn was lysed in lysis buffer-FOE (20 mM Tris-HCl, pH 7.5, 500 mM NaCl, 5 mM MgCl_2_, 0.01% CHAPS, 0.01% octyl-glcoside, 1 mM EDTA, 1 mM PMSF, 10% glycerol with Roche cOmplete protease inhibitor). After TEV cleavage, the eluted complex was subjected to anion exchange chromatography HiTrap Q HP 1 mL in the gradient of NaCl from 100 mM to 1 M. Peak fractions containing FACT complex was further purified in a size exclusion chromatography with Superdex 200 increase 10/300 GL. Purified complex was then analyzed by SDS-PAGE, silver staining, and western blotting. Anti-calmodulin binding protein epitope tag antibody (1:5000 dilution, Sigma) was used to detect Pob3 subunit by western blotting.

#### GST pulldown assay

BL21-CodonPlus competent cells were transformed with pGEX-6p-1 vectors expressing the fusion of GST-tag and fragments of *S. pombe* Mrc1 protein, including Mrc1-like domain, Pol1-N-terminal extension (NTE) and its mutants, *S. cerevisiae* Mrc1-like domain, or human Mrc1-like domain in CLASPIN connected by 3C protease cleavage site using protocols from Agilent. BL21-CodonPlus competent cells carrying pGEX-6p-1 vectors were cultured in 50 mL LB media with 100 μg/mL ampicillin and 25 μg/mL chloramphenicol and induced with 2% ethanol and 0.2 mM IPTG (AmericanBio) at 20 °C for 4 hours with shaking at 220 rpm starting with OD_600_=0.5~0.9. Cells are collected by centrifugation at 7,000 rpm for 10 min at 4 °C. Cell pellets were resuspended in B-PER Complete Bacterial Protein Extraction Reagent (ThermoFisher) supplemented with 900 mM NaCl, 1 mM PMSF, 1 mM DTT and 1 mM EDTA and lysed at 4 °C for 30 min with rotation. The lysate was then centrifuged at 15,000 rpm for 20 min at 4 °C. Supernatant was collected, diluted with one volume of Wash/Equilibrium buffer (20 mM HEPES-NaOH, pH 7.5, 500 mM NaCl, 0.02% Triton X-100, 1 mM EDTA, 1 mM DTT, 1 mM PMSF, 10% (v/v) glycerol) and incubated with 20 μL Pierce Glutathione Sepharose Magnetic Agarose Beads (ThermoFisher) at 4 °C for 1 h with rotation. The magnetic beads were collected on a magnetic stand and washed with Wash/Equilibrium buffer four times. To test the interaction between Mrc1-like domain and H3-H4 tetramer, FACT complex, the magnetic agarose beads was then equilibrated in the Binding buffer and incubated with in vitro reconstituted H3-H4 tetramer (Binding buffer for H3-H4 tetramer: 20 mM HEPES-NaOH pH 7.5, 500 mM NaCl, 0.02% Triton X-100, 1 mM EDTA, 1 mM DTT, 1 mM PMSF, 0.1 mg/mL insulin, 10% (v/v) glycerol) or purified fission yeast FACT (Binding buffer for FACT complex: 20 mM HEPES-KOH pH 7.5, 150 mM KOAc, 5 mM Mg(OAc)_2_, 1 mM EDTA, 1 mM EDTA, 10% (v/v) glycerol), which was endogenously expressed, at 4 °C for 1 h with rotation. For the GST-pulldown experiments to test the interaction between Pol1-NTE domain with FACT complex, H3-H4 tetramer and Mcl1-CTD domain, the wildtype and mutant GST-Pol1-NTE proteins were immobilized on the magnetic beads, equilibrated in the Pol1 binding buffer (PB buffer: 20 mM Tris-HCl, pH 7.5, 0.02% Triton X-100, 1 mM EDTA, 1 mM DTT, 1 mM PMSF, 0.1 mg/mL insulin, 10% (v/v) glycerol) and incubated with overexpressed fission yeast FACT in PB buffer + 100 mM NaCl, H3-H4 tetramer in PB buffer + 300 mM NaCl, or Mcl1-CTD domain in PB buffer + 150 mM NaCl at 4 °C for 1 h with mixing. The magnetic beads were collected on a magnetic stand and washed with Binding buffer for six times. The beads were then boiled in sample buffer and analyzed by SDS-PAGE and Coomassie blue stain, silver stain, and western blot.

#### Enrichment and sequencing of protein-associated nascent DNA (eSPAN)

The eSPAN assay in *S. cerevisiae* was adapted from previous methods with minor modifications^16,54^. *S. cerevisiae* yeast cells were cultured in YPD medium at 30°C and 180 rpm shaking until they reached the mid-log phase (OD_600_=0.4–0.5). To arrest cells at the G1 phase, they were treated with 5 μg/mL α-factor (Chinese Peptide Company) twice at 25°C and 180 rpm for one hour each time. Subsequently, the cells were pelleted by centrifugation at 2,500 rpm for 5 min at 4°C, washed three times with cold ddH_2_O, and then released into fresh YPD medium with 0.4 mg/mL BrdU (Sigma-Aldrich) at 23°C for 40 minutes to label newly synthesized DNA. Afterwards, the cells were crosslinked with 1% (w/v) paraformaldehyde (Sigma-Aldrich) at 25°C and with gentle rotation at 180 rpm for 20 minutes, followed by quenching with 125 mM glycine (Amresco) at 25°C and with gentle rotation at 180 rpm for 5 minutes.

The resulting cells were then pelleted, washed twice with cold TBS buffer (0.1 mM PMSF freshly added), and once with cold Buffer Z (1.2 M sorbitol, 50 mM Tris-HCl pH 7.4). The cells were resuspended in 8.7 mL Buffer Z (10 mM β-mercaptoethanol freshly added), and digested by adding 214 μL 5 mg/mL Zymolase (nacalai tesque) with incubation at 28°C and 100 rpm for approximately 35 minutes. The efficiency of digestion was checked by measuring the OD_600_ in 1% SDS, which should decrease to less than 10% of that pre-digestion value. The spheroplasts were collected by centrifugation, and the supernatant was aspirated. The pellet was gently resuspended in 1.5 mL of NP buffer (1 M sorbitol, 50 mM NaCl, 10 mM Tris-HCl pH 7.4, 5 mM MgCl_2_, 1 mM CaCl_2_, with 0.5 mM Spermidine, 0.007% (v/v) β-mercaptoethanol and 0.075% (v/v) NP-40 (Thermo) added freshly), and the resuspended pellet was divided into 4 equal parts, with each part containing 400 μL. The appropriate amount of MNase (Worthington, LS004797) was added to each part, and the reaction mixtures were incubated at 37 °C for 20 min to digest the chromatin into mainly mono- and di-nucleosome. The reaction was stopped by 8 μL 0.5 M EDTA (pH 8.0). Subsequently, 100 μL of 5× ChIP lysis buffer (250 mM HEPES-KOH pH 7.5, 700 mM NaCl, 5 mM EDTA pH 8.0, 5% (v/v) Triton X-100, 0.5% (w/v) Sodium deoxycholate, with 5 mM PMSF, 1.25 mg/mL pefabloc, 5 mg/mL bacitracin and 5 mM benzamidine added freshly) was added to the reaction mixtures, followed by 30 min of incubation on ice. The lysate was spun down twice at 10,800 rpm for 5 min and 15 min, respectively, at 4°C. The supernatant was collected and used for DNA extraction.

For each experiment, 50 μL of the supernatant was used as input, and 800 μL was used for ChIP against H3K4me3 or H3K56ac antibodies. For the ChIP assay, each sample was incubated with 0.6 ng anti-H3K4me3 antibody (Abcam) or 0.5 μL anti-H3K56ac antibody at 4°C for 12 hours, followed by incubation with 20 μL pre-washed protein G Sepharose agarose beads (GE Healthcare) at 4°C for 2 hours. The reaction mixtures were then washed extensively as below with 1 mL buffer per sample each time, and spun down at 2,500 rpm for 1 min at 4°C: 1xChIP lysis buffer (with 0.1 mM PMSF), once; 1xChIP lysis buffer, 5 min of incubation at 4°C, twice; 1xChIP lysis buffer (with 0.5 M NaCl), once; 1xChIP lysis buffer (with 0.5 M NaCl), 5 min of incubation at 4°C, once; Tris/LiCl buffer, once; Tris/LiCl buffer, 5 min of incubation at 4°C, once; Tris/EDTA buffer, twice. After washing, any remaining liquid was removed with fine syringe needles. Both the input and ChIP samples were reverse-crosslinked with chelex-100 (Bio-Rad). 50 μL 20% (w/v) chelex-100 is added to each sample, followed by 10 min at 100°C.

After cool-down, 5 μL 20 mg/mL Proteinase K (Invitrogen) was added, with 30 min of incubation at 55°C, followed by 10 min at 100 °C. The sample was then spun down at 14,000 rpm for 1 min and the supernatant was saved with 75 μL for the input sample and 25 μL for the ChIP sample. After adding 50 μL 2xTE to the original tube, the resulting DNA sample was cleared at 14,000 rpm for 1 min and mixed with the supernatant collected before. For the ChIP sample, 35 μL 1xTE was added to the original tube and cleared at 14,000 rpm for 1 min. The resulting 35 μL supernatant was saved and mixed with the supernatant collected before. For both the input and ChIP samples, 90 μL of the supernatant was used for BrdU-IP to obtain BrdU-IP and eSPAN samples, respectively.

For BrdU-IP, each sample was boiled at 100°C for 5 min and then snap-cooled in ice water for 5 min to get denatured single-stranded DNA. The reaction mixtures with anti-BrdU antibodies were prepared freshly with 800 μL cold BrdU-IP buffer (1xPBS, 0.0625% TritonX-100), 0.36 μL anti-BrdU antibody (BD Biosciences) and 0.3 μL 20 mg/mL *E. coli* tRNA (Roche) for each sample. The denatured sample was then mixed with 10 μL 10XPBS and 800 μL reaction mix, followed by 2 hours of incubation at 4°C. The reaction mixtures were then incubated with 15 μL pre-washed protein G beads for 2 hours at 4°C, followed with extensive wash as below: cold BrdU-IP buffer, 4–5 min of incubation at 4°C, three times; 1xTE, 4–5 min of incubation at room temperature, once. After washing, any remained liquid was removed with fine syringe needles. 100 μL elution buffer (1xTE, 1% (w/v) SDS) was added, followed by incubation for 15 min at 65°C with 1,300 rpm on Eppendorf Thermomixer C. The sample was then spun down at 14,000 rpm for 1 min and 85 μL supernatant was transferred to a new tube. Subsequently, 40 μL elution buffer was added, followed by incubation for 55 min at 65°C with 1,300 rpm. The sample was then spun down at 14,000 rpm for 1 min with 35 μL supernatant transferred and combined with the supernatant collected before. In total, six samples were obtained for each strain in one experiment: Input, MNase-BrdU-IP, H3K4me3-ChIP, H3K56ac-ChIP, H3K4me3-eSPAN, and H3K56ac-eSPAN. All the samples were purified using PCR MinElute Kit (Qiagen) to prepare DNA for library construction. Accel-NGS^™^ 1S Plus DNA Library Kit for Illumina^®^ (Swift) was applied to the ssDNA library. The ssDNA libraries were sequenced by Novogene Genome Sequencing Company with Illumina NovaSeq. The quality of samples was analyzed by real-time PCR. The primers used for qPCR quality control are listed in Table S1.

The eSPAN assay in *S. pombe* is described in^117^. All *S. cerevisiae* and *S. pombe* strains used for eSPAN experiments are listed in Table S1.

### QUANTIFICATION AND STAISTICAL ANALYSIS

#### Mass spectrometric data analysis

Mass spectra were processed using a Sequest-based in-house software pipeline. MS spectra were converted to mzXML using a modified version of ReAdW.exe. Database searching included all entries from *S. pombe*, which was concatenated with a reverse database composed of all protein sequences in reversed order. Searches were performed using a 50 ppm precursor ion tolerance. Product ion tolerance was set to 0.03 Th. Carbamidomethylation of cysteine residues (+57.0215Da) were set as static modifications, while oxidation of methionine residues (+15.9949 Da) was set as a variable modification.

Peptide spectral matches (PSMs) were altered to a 1% FDR^118,119^. PSM filtering was performed using a linear discriminant analysis, as described previously^120^, while considering the following parameters: XCorr, ΔCn, missed cleavages, peptide length, charge state, and precursor mass accuracy. Peptide-spectral matches were identified, quantified, and collapsed to a 1% FDR and then further collapsed to a final protein-level FDR of 1%. Furthermore, protein assembly was guided by principles of parsimony to produce the smallest set of proteins necessary to account for all observed peptides.

#### eSPAN sequencing analysis

After quality control, Trimmomatic was used to remove the adaptor and discard sequencing reads with low-quality^121^. The clean reads were then mapped to the yeast reference genome sacCer3 using Bowtie2^122^. Only paired-end reads that were correctly mapped on both ends were selected \for further analysis. Each read was assigned to the Watson or Crick strand based on the flag in the SAM files. BrdU-enriched regions were defined with MACS2^123^, and DANPOS was used to call nucleosome positions and occupancy^124^. The final eSPAN density at Watson or Crick strand was normalized by MNase-BrdU-IP-ssSeq data. eSPAN data were analyzed by calculating the log2 ratio between normalized eSPAN signal at the Watson strand and the normalized eSPAN signal at the Crick strand. The significance of the difference of histone inheritance at the leading strand or lagging strand was tested by the Wilcoxon test.

## Supplementary Material

1 **Figure S1. Mutations in replisome components abolish the maintenance of heterochromatin and H3K9 methylation, related to** Figure 1. A) Diagram of the replication fork showing the location of replisome components that have reported histone binding activity. B) Heterochromatin maintenance assay showing the maintenance phenotype of cells carrying mutations that have reduced histone binding activities in vitro. *mcm2-3A* denotes *mcm2* with E77A, Y80A, Y89A amino acid substitutions reported in^32^. *pol1-6A* denotes *pol1* with Y40A, Y48A, F61A, D65A, G69A, Y70A amino acid substitutions reported in^35^. C, D) H3K9me2 ChIP-qPCR at the *10XtetO-ade6*^+^ locus showing H3K9me2 levels in *mcm2*^+^ or *mcm2-3A* cells at the establishment phase (C, AHT−) and the maintenance phase 24 hours after growth in the presence of AHT (D, AHT+). E) Diagram showing the yeast FACT complex subunits (Spt16, Pob3 and accessory factor Nhp6), SMARCAD1 family ATPase Fft3, and CAF-1 complex subunits (Pcf1, Pcf2, Pcf3). F) Heterochromatin maintenance assay showing the epigenetic inheritance phenotypes of cells lacking the non-essential replication-associated histone chaperone subunits. G) Diagram illustrating the domains in Mrc1. The locations of nonsense Mrc1 mutations are highlighted in red. Previously reported amino acids of Mrc1 involved in mediating replication checkpoint signaling are highlighted in a pink box as the SQ/TQ domains. The location of the SQ, TQs and nonsense mutations isolated from the genetic screen are highlighted below the cartoon diagram of Mrc1. The location of the *S. pombe* Mrc1 phosphodegron motif and phosphodegron mutant (Mrc1-SSAA) are also indicated below the diagram. H) Diagram illustrating the conserved replication checkpoint pathway involving the upstream checkpoint kinase Rad3, mediator for replication checkpoint Mrc1, and downstream checkpoint effector Cds1. I) Heterochromatin maintenance assay showing the maintenance phenotypes of cells carrying replication checkpoint deficient *mrc1* alleles or cells lacking the checkpoint effector Cds1. J) Western blots showing that compared to wild-type Mrc1 protein, the expression level of Mrc1-SSAA is similar to Mrc1-(1–620) (expressed from *mrc1-W620STOP)* and Mrc1-(1–769) (expressed from *mrc1-K769STOP)*. K) Heterochromatin maintenance assay showing the maintenance phenotypes of cells carrying *mrc1-SSAA*, epitope tagged *mrc1-SSAA* alleles, or TAP-tagged *sld5* allele. L) IP-MS analysis of FLAG-tagged heterochromatin maintenance competent Mrc1-SSAA and heterochromatin maintenance deficient mutant Mrc1-(1–769). Colors indicate proteins in the complexes shown below the plot.

2 **Figure S2. Quality control of the AlphaFold-Multimer predicted structures of *S. pombe* Mrc1-like domain in complex with (H3.1-H4)**_**2**_**, related to** Figure 2. A) Predicted aligned error (PAE) plots of all five rank AlphaFold models of the Mrc1-like domain (H3.1-H4)_2_ structures. Low aligned error (PAE<10) between two amino acids implies restrictions resulting from possible protein-protein interactions. B) AlphaFold-predicted local distance difference score (pLDDT) of all amino acids of Mrc1-like domain in all five predicted model of Mrc1-like domain (H3.1-H4)_2_. pLDDT < 50 suggests very low confidence prediction, 50<pLDDT<70 suggests low confidence prediction, 70<pLDDT<90 suggests confident prediction, and pLDDT>90 suggests very high confidence prediction. C) PAE plots of all five predicted structures of full-length Mrc1-(H3-H4)_2_. D) The first rank predicted structure of Mrc1-(Cnp1-H4)_2_. E) The pLDDT map of the Mrc1-like domain in the predicted structure of Mrc1-(Cnp1-H4)_2_. F) The PAE plot for the predicted structure of Mrc1-(Cnp1-H4)_2_. G) AlphaFold-Multimer predicted structures of Mrc1-like domain from *S. pombe* Mrc1 and its homologs in the indicated organisms interacting with histone H3.1-H4 tetramer in eukaryotes. Bottom row shows a phylogenetic tree of the nine eukaryotic species used for comparative structural analysis.

3 **Figure S3. Structural analysis of histone binding activities of replisome components predicted by AlphaFold-Multimer, related to** Figure 2. A) Top, diagram illustrating the location of Mcm2 histone binding domain (HBD) at the N-terminal extension of Mcm2. Bottom, predicted structures of *S. pombe* Mcm2-HBD with H3.1-H4 tetramer, modified crystal structure of human MCM2-HBD with H3.3-H4 tetramer (PDB: 5BNV)^33^, and alignment of the two structures. Conserved amino acids involved in histone binding and heterochromatin maintenance (Figure S2B) are highlighted in the structure. B) Top, Diagram illustrates predicted Spt16 histone interaction domains. Bottom, predicted structure of Spt16-middle domain and C-terminal domain (MD/CTD) interacting with H3.1-H4 tetramer, published crystal structure of human SPT16-(MD/CTD) with H3.1-H4 tetramer (PDB:4Z2M)^46^ and alignment of the two structures. C) Top, Diagram illustrates the regions at the N-terminal extension (NTE) of Pol1 predicted by AlphaFold. The α2 helix, predicted to bind to histone H3-H4, is highlight in magenta color. Middle, the predicted structure of Pol1(NTE)-H3.1-H4. The amino acids that are conserved and required for heterochromatin maintenance (Figure S2B) are highlighted in the model. Bottom, the PAE plot of the predicted structure of *S. pombe* Pol1(NTE) with the H3-H4 tetramer. D) Top, the domains in *S. pombe* histone-like proteins Dpb3 and Dpb4 predicted by AlphaFold-Multimer. Middle, the predicted structure of *S. pombe* Dpb3-Dpb4-H3.1-H4 tetramer. Bottom, the PAE plot of the predicted structure of Dpb3-Dpb4-H3-H4 tetramer. E) Top, the domains in human MCM10 predicted by AlphaFold-Multimer. Middle, predicted structure of human MCM10-H3.1-H4 tetramer. Bottom, the PAE plot of the predicted structure of human MCM10-H3.1-H4 tetramer. F) Left, the crystal structure of nucleosome core particle (PDB: 1AOI)^26^ used for alignment. Right, the predicted structure of Mrc1-like domain-(H3.1-H4)_2_ used for alignment. G) Alignment of predicted structure of Mrc1-like domain with (H3.1-H4)_2_ and the crystal structure of nucleosome core particle shows the locations of α1–3 of Mrc1-like domain in the predicted structure relative to the location of nucleosomal DNA (from Dyad to SHL-3), histone H2B-α2 and histone H2A-C-terminal extension (CTE), respectively. The α2–3 of Mrc1-like domain tilts ~10.8°, compared to H2B-α2 and H2A-CTE. For illustration purposes, only the relevant regions of nucleosomal DNA and histones are shown.

4 **Figure S4. Interactions between Mrc1-like domain and histones, related to** Figure 3. A) PAE plots of predicted interaction between *S. pombe* Mrc1-like domain and H2A/H2B dimer. B) GST-Mrc1-like domain protein from *S. pombe* binds H2A-H2B weakly under stringent binding conditions (500 mM NaCl). C) GST-Mrc1-like domain fusion proteins from *S. pombe* and human pull down histone H3-H4 under stringent binding conditions (500 mM NaCl). D) GST pull-down assays showing that the interaction between *S. cerevisiae* Mrc1-like domain and H3-H4 is salt-sensitive. E) SEC-MALS distribution of Mrc1-(651–900)-(H3-H4)_2_ complex. Black curve indicates cumulative molar mass in the range of the indicated molar mass, and red curve indicates linear differential molar mass at the indicated molar mass. F) Diagram summarizing mutations of the Mrc1-like domain that specifically abolish heterochromatin maintenance isolated from targeted mutagenesis or generated based on structural predictions. G) Bar plot showing the percentage of red or variegated cells that maintain heterochromatin in the indicated *mrc1* mutant cells in panel E; *mrc1-3A (mrc1-M755A,F758A,L774A)*. n=3. Error bars indicate the standard deviation.

5 **Figure S5. Role of Mrc1 and its histone binding domain in parental histone transfer in *S. cerevisiae* and *S. pombe*, related to** Figure 5. A) Diagram illustrates the workflow of eSPAN analysis of histone occupancy at nascent chromatin in S phase. The right panels illustrate the interpretation of eSPAN bias and the expected outcomes of leading or lagging strand biases of protein occupancy at the nascent chromatin. B) Bar plot showing the distribution of eSPAN bias of H3K4me3 between the left and right side of the 139 early ACS regions in the *S. cerevisiae* eSPAN samples shown in Figure 5D. P-values were determined by Wilcoxon rank-sum test. C) eSPAN density of parental histones (H3K4me3) at the leading strands and lagging strands in the *S. cerevisiae* eSPAN samples shown in Figure 5D. Statistical significance test at the leading strands between *MRC1* and *mrc1-ΔHBD* cells: p = 2.5e-7, Wilcoxon rank-sum test. D) Bar plot showing the distribution of eSPAN bias of H3K56ac between the left and right side of the 139 early ACS regions in the *S. cerevisiae* eSPAN samples shown in Figure 4F. E) Second biological replicates of eSPAN bias of parental histone surrogate H3K4me3 in *MRC1* wild-type, *mrc1-α2*Δ cells. F-G) Two biological replicates of eSPAN bias of new histone surrogate H3K56ac in *MRC1* wild-type, *mrc1-α2*Δ cells H-I) Heatmap presentations of eSPAN bias of parental histones H3K4me3 (H) and newly deposited H3K56ac (I) among 139 early replicating ACSs in *MRC1* wild-type, *mrc1*Δ, *mrc1-like domainΔ* (amino acid 711 to 850), and two replicates of *mrc1-α2Δ S. cerevisiae* cells. J) Heatmap presentations of eSPAN bias of parental histone (H3K4me3) among 162 early replicating ACSs in *mrc1*^+^, *mrc1*-*3A* (two biological replicates), and *mcm2-2A S. pombe* cells.

6 **Figure S6. Structural predictions suggest interactions between FACT and replisome components, related to** Figure 6. A) IP-MS analysis of TAP-tagged Sld5 from *mrc1*^+^ and *mrc1-3A* cells. Colors indicate replisome components shown below the plot. B) Heatmap showing the average interface predicted template modeling (ipTM) score of all five predicted models between *S. pombe* FACT subunits and each core replisome component. The ipTM and heatmap scale ranges from 0.25 to 0.7. Although the ipTM score for the Spt16 and Pri1 interaction is high, the interaction interface appears to be small and clashes with the interaction interface between Pri1 and Pri2 in the published cryo-EM primase structure (PDB: 8B9C)^104^. C) In vitro GST pull-down assays using the indicated GST-Mrc1 segments to pull down endogenously purified FACT. SDS-PAGE gels show purified FACT (left), input (middle), and bound fractions (right). D) PAE of the AlphaFold-Multimer predicted interaction between Spt16-Mrc1. E) Based on the GST pull-down results, the first interaction between Mrc1 and FACT localizes at the N-terminus of Mrc1 (FBD1, amino acids 134–168) and the Spt16-middle domain. The location of other binding domains is shown for reference. F) the predicted structure of Mrc1-FBD1 in complex with Spt16-DD-MD and H3-H4 tetramer. G) The PAE plot of the predicted structure in panel F. H) The second interaction interface between Spt16 and Mrc1 localizes to a middle region of Mrc1 (Mrc1-FBD2, amino acids 513–540) and the N-terminal domain of Spt16 (Spt16-NTD). I) the predicted structure of Spt16 (without CTD)-Mrc1(middle region including FBD2)-H3-H4 tetramer. J) The PAE plot of the predicted structure in panel I.

7 **Figure S7. Mapping the locations of FACT and parental histones on the cryo-EM structure of the replisome, related to** Figure 6. A) PAE plot of the predicted structure of Swi1 and Spt16 interaction shown in Figure 7A–B. B) PAE plot of the predicted structure of *S. pombe* Mcm2(NTE)-Swi1(NTD)-Spt16(DD-MD)-(H3-H4)_2_ shown in Figure S7D. C) Summary of the NTEs of Mcm2 and Mrc1 predicted to interact with Swi1. D) Published cryo-EM structure (PDB: 7PFO) of human N-terminal CLASPIN bound to TIMELESS. E) Predicted structure of *S. pombe* N-terminal Mrc1 and Mcm2 bound to Swi1. F) PAE plot of the predicted structure shown in panel 6E. G) -I) Predicted structure in panel 6E with highlighted interaction between G) Spt16 and Pol1, H) H3-H4 and Pol1, I) Mcl1-CTD and Pol1. J) Diagrams indicating the domains of Cdc45, Mcm2 and Mrc1 shown in Figure S7K–L. K) Published cryo-EM structure (PDB: 8B9C) of *S. cerevisiae* Cdc45, N-terminal domain of Mcm2 and Mrc1-like domain interaction. L) Predicted structure of *S. cerevisiae* Mrc1-like domain with (H3.1-H4)_2_, Cdc45 and N-terminal domain of Mcm2. M) Diagram indicating the *S. pombe* Mrc1-like domain. N) Predicted structure of *S. pombe* Mrc1-like domain with (H3.1-H4)_2_, Cdc45 and N-terminal domain of Mcm2. O) PAE plot of the predicted structure shown in panel L. P) PAE plot of the predicted structure shown in panel N.

8 **Figure S8. Structural predictions suggest that the Mrc1 histone binding domain can bind H3-H4 tetramer together with other replisome histone binding domains, related to** Figure 7. A) Predicted structure of *S. pombe* Mrc1-like domain with (H3.1-H4)_2_ and Mcm2. B) Rank 1 PAE plot of the predicted structure in panel A. C) Predicted structure of *S. pombe* Mrc1-like domain with (H3.1-H4)_2_ and Pol1. D) Rank 1 PAE plot of the predicted structure in panel C. E) Predicted structure of *S. pombe* Mrc1-like domain with (H3.1-H4)_2_ and Dpb3/Dpb4. F) Rank 1 PAE plot of the predicted structure in panel E. The confidence of the interaction between Dpb3-Dpb4 and (H3.1-H4)_2_ is lower compared to all other replisome histone binding components.

9 **Table S1 - List of yeast strains and oligos used in this study. Related to** STAR Methods.

10 **Table S2 – List of associated proteins in Mrc1-TAP, truncated Mrc1-TAP immunoprecipitation samples identified by mass spectrometry. Related to** Figure 1.

11 **Table S3 – List of proteins in Mrc1-FLAG and truncated Mrc1-FLAG immunoprecipitation samples identified by mass spectrometry. Related to** Figure 1.

12 **Table S4 – List of proteins in TAP-Sld5 immunoprecipitations from *mrc1***^**+**^ ***and mrc1-W620X* cells identified by mass spectrometry. Related to** Figure 1.

13 **Table S5 – List of proteins in TAP-Sld5 immunoprecipitations from *mrc1***^**+**^ ***and mrc1-3A* cells identified by mass spectrometry. Related to** Figure 4.

14 **Table S6 - List of AlphaFold-Multimer predictions in this study. Related to** STAR Methods.

## Figures and Tables

**Table:** KEY RESOURCES TABLE

REAGENT or RESOURCE	SOURCE	IDENTIFIER
**Antibodies**		
Mouse monoclonal anti-H3K9me2	Abcam	Cat# ab1220; RRID:AB_449854
Rabbit polyclonal peroxidase anti-peroxidase soluble complex antibody	Sigma-Aldrich	Cat# P1291; RRID:AB_1079562
Mouse monoclonal anti-FLAG M2	Sigma-Aldrich	Cat# F1804; RRID:AB_262044
Rabbit polyclonal anti-calmodulin binding protein epitope tag	Sigma-Aldrich	Cat# 07–482; RRID:AB_310653
Rabbit polyclonal anti-H3K4me3	Abcam	Cat# ab8580; RRID:AB_306649
Mouse monoclonal anti-BrdU	BD Biosciences	Cat# 555627
Rabbit polyclonal anti-H3K56ac	This study	N/A
IgG from rabbit serum	Sigma-Aldrich	Cat# I5006; RRID:AB_1163659
**Chemicals, peptides, and recombinant proteins**		
Anhydrotetracycline (hydrochloride)	Cayman chemical	Cat# 10009542
G418 sulfate	Thermo Fisher Scientific	Cat# 11811031
clonNAT	Werner BioAgents	Cat# 5002000
Hygromycin B	Sigma-Aldrich	Cat# 10843555001
Blasticidin S HCl	GoldBio	Cat# B-800
Hydroxyurea	Sigma-Aldrich	Cat# H8627
EMM powder	Sunrise Science Products	Cat# 2005
5-FOA	Goldbio	Cat# F-230
PMSF Protease Inhibitor	Thermo Fisher Scientific	Cat# 36978
cOmplete^™^, EDTA-free Protease Inhibitor Cocktail	Sigma-Aldrich	Cat# COEDTAF-RO
protease inhibitor cocktail	Sigma-Aldrich	Cat# P8215
Dynabeads^™^ Protein A	Invitrogen	Cat# 10002D
Dynabeads^™^ Protein G	Invitrogen	Cat# 10004D
Dynabeads^™^ M-270 Epoxy	Invitrogen	Cat# 14302D
DMP (dimethyl pimelimidate)	Thermo Fisher Scientific	Cat# 21667
Ethanolamine	Sigma-Aldrich	Cat# E9508
Benzonase	Santa Cruz Biotechnology	Cat# sc-391121C
Pierce^™^ 16% Formaldehyde (w/v), Methanol-free	Thermo Fisher Scientific	Cat# 28908
Proteinase K, recombinant, PCR Grade	Sigma-Aldrich	Cat# RPROTKSOL-RO
Phenol:Chloroform:Isoamyl Alcohol 25:24:1 Saturated with 10 mM Tris, pH 8.0, 1 mM EDTA	Sigma-Aldrich	Cat# P2069
Glycogen	Sigma-Aldrich	Cat# 10901393001
Rabbit IgG HRP Linked Whole Ab	Sigma-Aldrich	Cat# GENA934-1ML
Mouse IgG HRP Linked Whole Ab	Sigma-Aldrich	Cat# GENA931-1ML
4–15% Mini-PROTEAN^®^ TGX^™^ Precast Protein Gels, 15-well, 15 μl	Bio-rad	Cat# 4561086
Terrific Broth Modified	US Biological	Cat# T15050–5000.0
IPTG	AmericanBio	Cat# AB00841-00050
B-PER Complete Bacterial Protein Extraction Reagent	Thermo Fisher Scientific	Cat# 89821
Glutathione Sepharose 4 Fast Flow GST-tagged protein purification resin	Cytiva	Cat# 17513202
Pierce^™^ Glutathione Magnetic Agarose Beads	Thermo Fisher Scientific	Cat# 78601
Insulin	Sigma-Aldrich	Cat# I9278
IgG Sepharose 6 Fast Flow affinity resin	Cytiva	Cat# 17096901
Taq DNA polymerase	This study	N/A
H3-H3 tetramer	This study	N/A
H2A-H2B dimer	This study	N/A
3C protease	This study	N/A
TEV protease	This study	N/A
DTT	Sigma-Aldrich	Cat# 10708984001
TCEP	Gold Biotechnology	Cat# TECP2
BSA	Thermo Scientific	Cat# 23209
EPPS	Sigma-Aldrich	Cat# E9502
Urea	Sigma-Aldrich	Cat# U5378
Trypsin	Promega	Cat# V511C
alpha-Mating Factor Pheromone, yeast	Chinese peptide company	Cat# SIGN-001
Paraformaldehyde (1%, w/v)	Sigma-Aldrich	Cat# P6148–1KG
Glycine	Amresco	Cat# 0167–5KG
BrdU	Sigma-Aldrich	Cat# B5002–5G
Zymolyase-100T	nacalai tesque	Cat# 07665–84
NP-40	Thermo Fisher Scientific	Cat# 28324
Nuclease, Micrococcal (MNase)	Worthington	Cat# LS004797
Protein G Sepharose agarose beads	GE Healthcare	Cat# 17061801
Chelex-100	Bio-rad	Cat# 1422822
*E. coli* tRNA	Roche	Cat# 10109541001
CHAPS	anatrace	Cat# C316S
Octyl-glucoside	anatrace	Cat# O311S
**Critical commercial assays**		
Invitrogen SimplyBlue^™^ SafeStain	Thermo Fisher Scientific	Cat# LC6065
Pierce^™^ Silver Stain Kit	Thermo Fisher Scientific	Cat# 24612
SuperSignal^™^ West Pico PLUS Chemiluminescent Substrate	Thermo Fisher Scientific	Cat# 34580
millTUBE 1 mL AFA fiber	Covaris	Cat# 520130
No. 1.5H high precision glass coverslips (24×50 mm)	Thorlabs	Cat# CG15KH
Qiagen MinElute Kit	Qiagen	Cat# 28004
Accel-NGS^™^ 1S Plus DNA Library Kit for the Illumina^®^	Swift	Cat# 10096
**Experimental models: Organisms/strains**		
*S. pombe* strains	This study	Table S1
*S. cerevisiae* strains	This study	Table S1
*E. coli* BL21-CodonPlus (DE3)-RIPL strain	Agilent	Cat# 230280
**Oligonucleotides**		
gRNAs for genome editing	This study	Table S2
qPCR Primers	This study	Table S2
**Deposited data**		
Immunoprecipitation-coupled mass spectrometry	This study	Table S3
Predicted structures by AlphaFold-Multimer	This study	Model Archive: ma-dm-hisrep; Table S4
Raw and processed eSPAN data	This study	Project: PRJCA018248; GRA: CRA011810; CRA014983; GSE269383
**Software and algorithms**		
UCSF Chimera X daily build (2022-10-26) version	UCSF Chimera X	RRID:SCR_015872
ColabFold	Google Colab	N/A
localColabFold	Harvard Medical School O2 computing cluster	N/A
In-house mass spectrometry data analysis software	109	N/A
ChatGPT3.5 (March 24 version)	OpenAI	RRID:SCR_023775
Clustal Omega	UniProt	RRID:SCR_001591
JalView	University of Dundee	RRID:SCR_006459
AcquireMP	Refeyn, Ltd	N/A
DiscoverMP	Refeyn, Ltd	N/A
ASTRA, version 7.3.2.21	Wyatt	RRID:SCR_016255
Bowtie2	John Hopkins University	RRID:SCR_016368
MACS	Dana Farber Cancer Institute	RRID:SCR_013291
DANPOS	Baylor College of Medicine	RRID:SCR_015527
**Other**		
MagNA Lyser Instrument	Roche	Cat# 3358968001
QuantStudio^™^ 7 Flex Real-Time PCR System, 384-well, desktop	Applied Bioystems	Cat# 4485701
6875 Freezer/Mill^®^ High Capacity Cryogenic Grinder	SPEXSamplePrep	Cat# 6875
E220evolution Focused-ultrasonicator	Covaris	Cat# 500429
Q Exactive HF-X Hybrid Quadrupole-Orbitrap MS System	Thermo Fisher Scientific	Cat# 0726042
Accela 600 Pump	Thermo Fisher Scientific	Cat# 6003–0160
Accucore^™^ C18 HPLC Columns	Thermo Fisher Scientific	Cat# 17126–032130
Refeyn TwoMP mass photometry	Refeyn, Ltd	N/A
HiTrap Q HP 1 mL	Cytiva	Cat# 17115301
Amicon 10 MWCO Ultra-4 Centrifugal Filter Unit	Sigma-Aldrich	Cat# UFC8010
Superdex 200 increase 10/300 GL	Sigma-Aldrich	Cat# GE28-9909-44
Superdex 200 increase 3.2/300	Cytiva	Cat# 28990946
Agilent 1260 Infinity LC System with UV detector	Agilent	RRID:SCR_019511
Wyatt Dawn Heleos II MALS detector	Wyatt	RRID:SCR_020896
Wyatt Optilab T-rEX Refractive Index Detector	Wyatt	N/A

## Highlights

Mrc1/CLASPN and other replisome subunits are required for heterochromatin inheritance

Mrc1 forms a brace around a histone H3-H4 tetramer mimicking nucleosome features

Mrc1 mediates parental histone transfer to both newly synthesized daughter DNA strands

Mrc1 may act as a distribution site in the replisome for parental histone transfer
