# Supplementary figures and images for: A replisome-associated histone H3-H4 chaperone required for epigenetic inheritance
Source: Cell. Author manuscript; Available in PMC 2024 Sep 8. (PMC11380579; doi:10.1016/j.cell.2024.07.006)

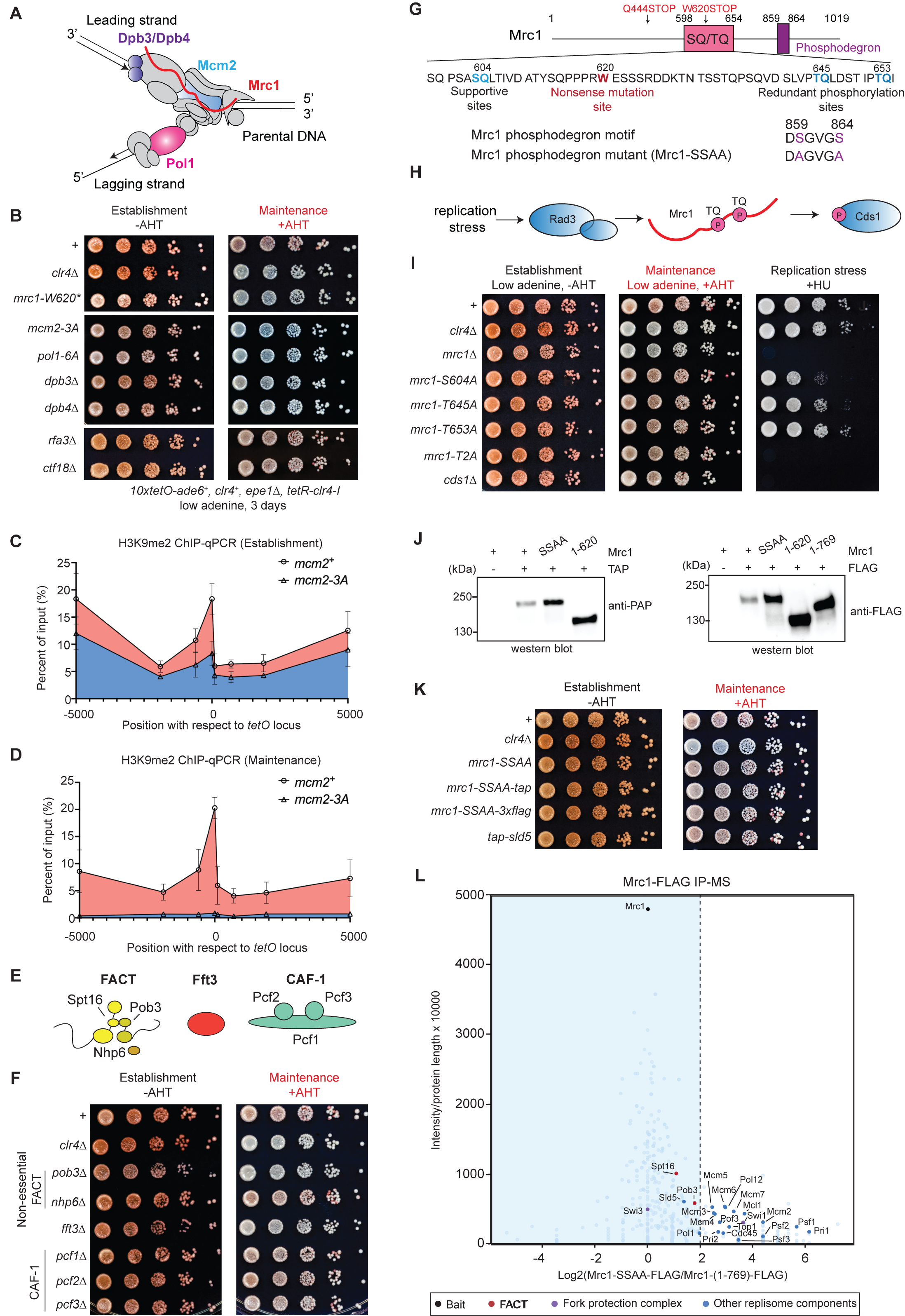

Supplement: 1 — Figure S1. Mutations in replisome components abolish the maintenance of heterochromatin and H3K9 methylation, related to Figure 1. A) Diagram of the replication fork showing the location of replisome components that have reported histone binding activity. B) Heterochromatin maintenance assay showing the maintenance phenotype of cells carrying mutations that have reduced histone binding activities in vitro. mcm2-3A denotes mcm2 with E77A, Y80A, Y89A amino acid substitutions reported in32. pol1-6A denotes pol1 with Y40A, Y48A, F61A, D65A, G69A, Y70A amino acid substitutions reported in35. C, D) H3K9me2 ChIP-qPCR at the 10XtetO-ade6+ locus showing H3K9me2 levels in mcm2+ or mcm2-3A cells at the establishment phase (C, AHT−) and the maintenance phase 24 hours after growth in the presence of AHT (D, AHT+). E) Diagram showing the yeast FACT complex subunits (Spt16, Pob3 and accessory factor Nhp6), SMARCAD1 family ATPase Fft3, and CAF-1 complex subunits (Pcf1, Pcf2, Pcf3). F) Heterochromatin maintenance assay showing the epigenetic inheritance phenotypes of cells lacking the non-essential replication-associated histone chaperone subunits. G) Diagram illustrating the domains in Mrc1. The locations of nonsense Mrc1 mutations are highlighted in red. Previously reported amino acids of Mrc1 involved in mediating replication checkpoint signaling are highlighted in a pink box as the SQ/TQ domains. The location of the SQ, TQs and nonsense mutations isolated from the genetic screen are highlighted below the cartoon diagram of Mrc1. The location of the S. pombe Mrc1 phosphodegron motif and phosphodegron mutant (Mrc1-SSAA) are also indicated below the diagram. H) Diagram illustrating the conserved replication checkpoint pathway involving the upstream checkpoint kinase Rad3, mediator for replication checkpoint Mrc1, and downstream checkpoint effector Cds1. I) Heterochromatin maintenance assay showing the maintenance phenotypes of cells carrying replication checkpoint deficient mrc1 al [file NIHMS2018534-supplement-1.tif]

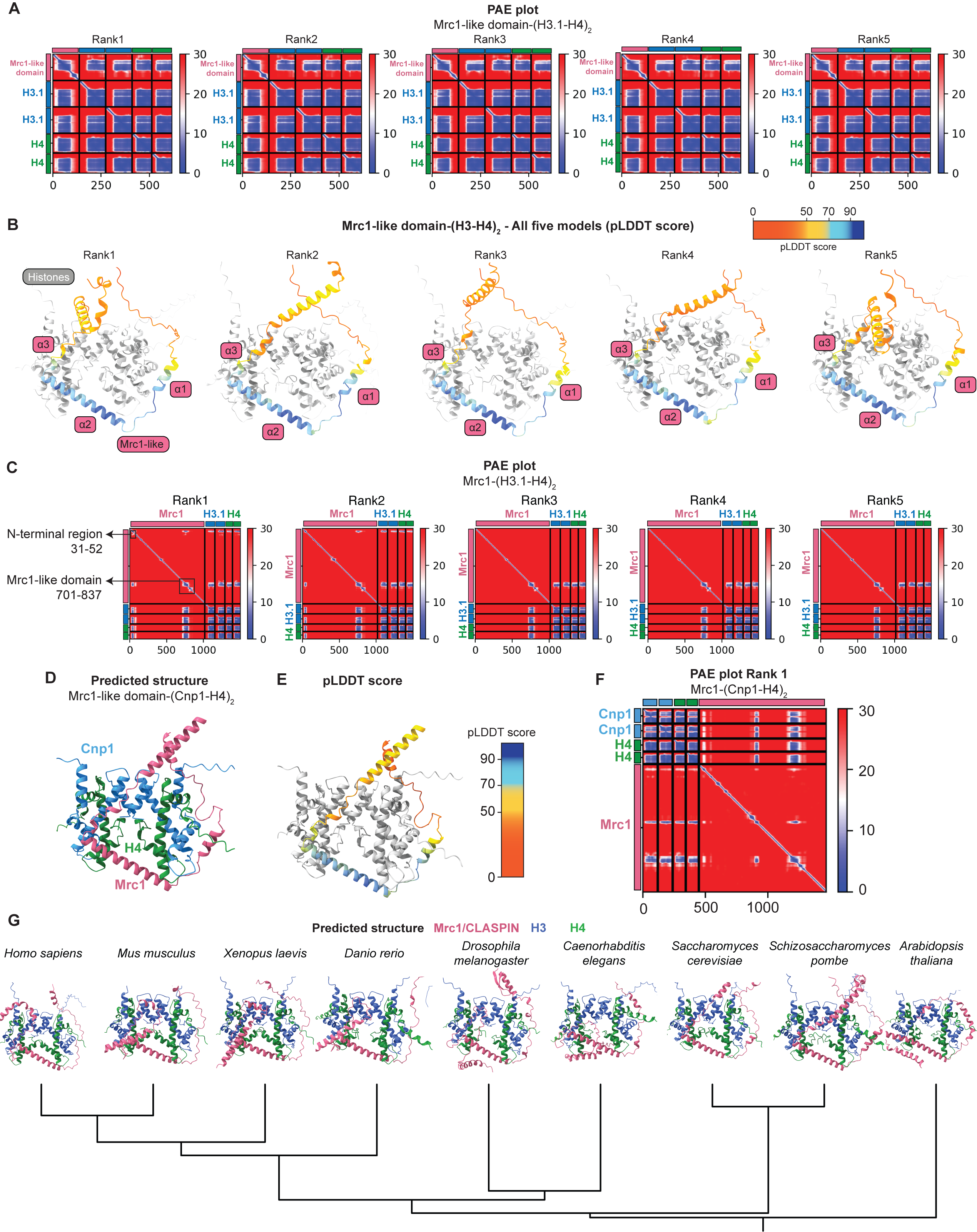

Supplement: 2 — Figure S2. Quality control of the AlphaFold-Multimer predicted structures of S. pombe Mrc1-like domain in complex with (H3.1-H4)2, related to Figure 2. A) Predicted aligned error (PAE) plots of all five rank AlphaFold models of the Mrc1-like domain (H3.1-H4)2 structures. Low aligned error (PAE<10) between two amino acids implies restrictions resulting from possible protein-protein interactions. B) AlphaFold-predicted local distance difference score (pLDDT) of all amino acids of Mrc1-like domain in all five predicted model of Mrc1-like domain (H3.1-H4)2. pLDDT < 50 suggests very low confidence prediction, 50<pLDDT<70 suggests low confidence prediction, 70<pLDDT<90 suggests confident prediction, and pLDDT>90 suggests very high confidence prediction. C) PAE plots of all five predicted structures of full-length Mrc1-(H3-H4)2. D) The first rank predicted structure of Mrc1-(Cnp1-H4)2. E) The pLDDT map of the Mrc1-like domain in the predicted structure of Mrc1-(Cnp1-H4)2. F) The PAE plot for the predicted structure of Mrc1-(Cnp1-H4)2. G) AlphaFold-Multimer predicted structures of Mrc1-like domain from S. pombe Mrc1 and its homologs in the indicated organisms interacting with histone H3.1-H4 tetramer in eukaryotes. Bottom row shows a phylogenetic tree of the nine eukaryotic species used for comparative structural analysis. [file NIHMS2018534-supplement-2.tif]

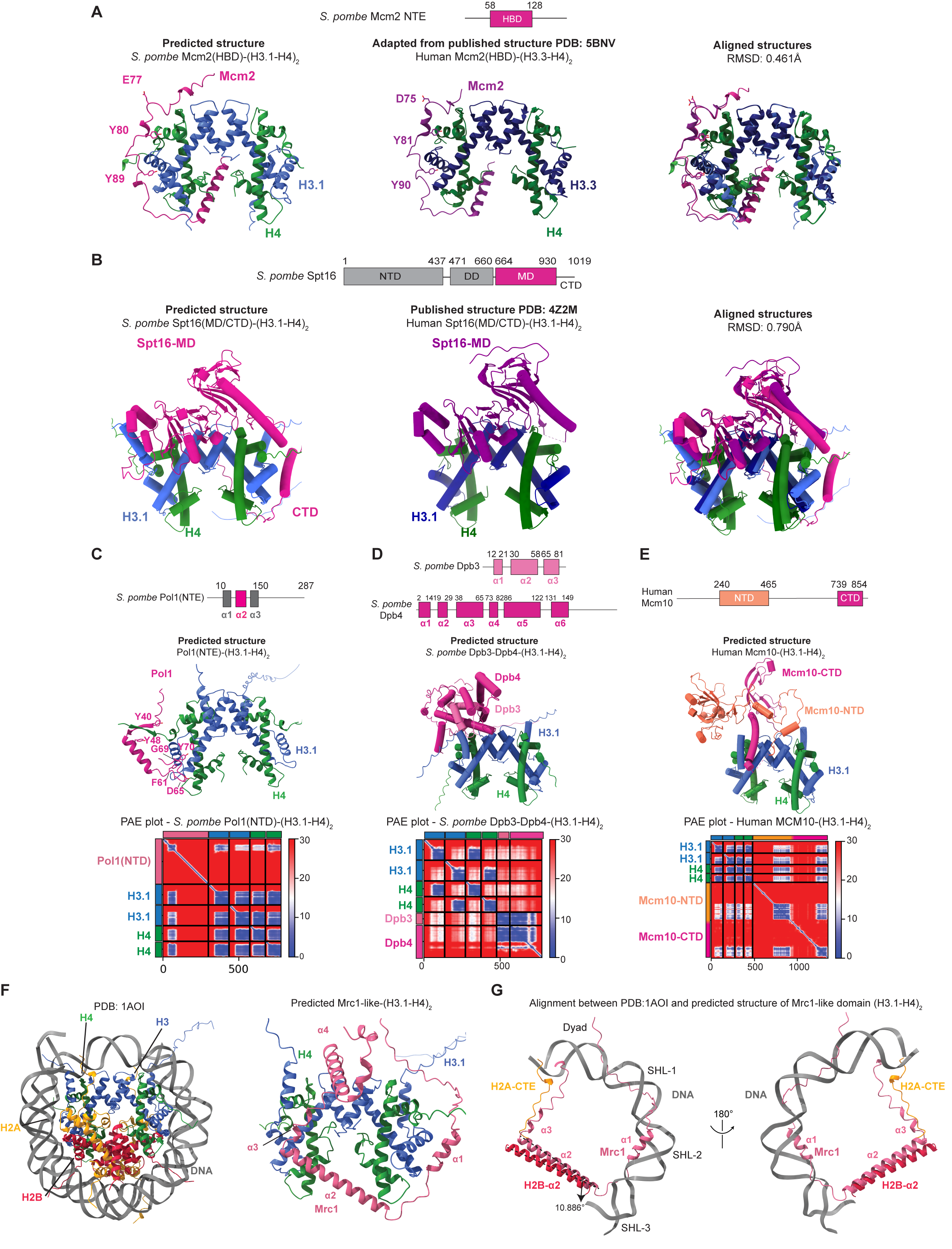

Supplement: 3 — Figure S3. Structural analysis of histone binding activities of replisome components predicted by AlphaFold-Multimer, related to Figure 2. A) Top, diagram illustrating the location of Mcm2 histone binding domain (HBD) at the N-terminal extension of Mcm2. Bottom, predicted structures of S. pombe Mcm2-HBD with H3.1-H4 tetramer, modified crystal structure of human MCM2-HBD with H3.3-H4 tetramer (PDB: 5BNV)33, and alignment of the two structures. Conserved amino acids involved in histone binding and heterochromatin maintenance (Figure S2B) are highlighted in the structure. B) Top, Diagram illustrates predicted Spt16 histone interaction domains. Bottom, predicted structure of Spt16-middle domain and C-terminal domain (MD/CTD) interacting with H3.1-H4 tetramer, published crystal structure of human SPT16-(MD/CTD) with H3.1-H4 tetramer (PDB:4Z2M)46 and alignment of the two structures. C) Top, Diagram illustrates the regions at the N-terminal extension (NTE) of Pol1 predicted by AlphaFold. The α2 helix, predicted to bind to histone H3-H4, is highlight in magenta color. Middle, the predicted structure of Pol1(NTE)-H3.1-H4. The amino acids that are conserved and required for heterochromatin maintenance (Figure S2B) are highlighted in the model. Bottom, the PAE plot of the predicted structure of S. pombe Pol1(NTE) with the H3-H4 tetramer. D) Top, the domains in S. pombe histone-like proteins Dpb3 and Dpb4 predicted by AlphaFold-Multimer. Middle, the predicted structure of S. pombe Dpb3-Dpb4-H3.1-H4 tetramer. Bottom, the PAE plot of the predicted structure of Dpb3-Dpb4-H3-H4 tetramer. E) Top, the domains in human MCM10 predicted by AlphaFold-Multimer. Middle, predicted structure of human MCM10-H3.1-H4 tetramer. Bottom, the PAE plot of the predicted structure of human MCM10-H3.1-H4 tetramer. F) Left, the crystal structure of nucleosome core particle (PDB: 1AOI)26 used for alignment. Right, the predicted structure of Mrc1-like domain-(H3.1-H4)2 used for alignment. G) Alignment of [file NIHMS2018534-supplement-3.tif]

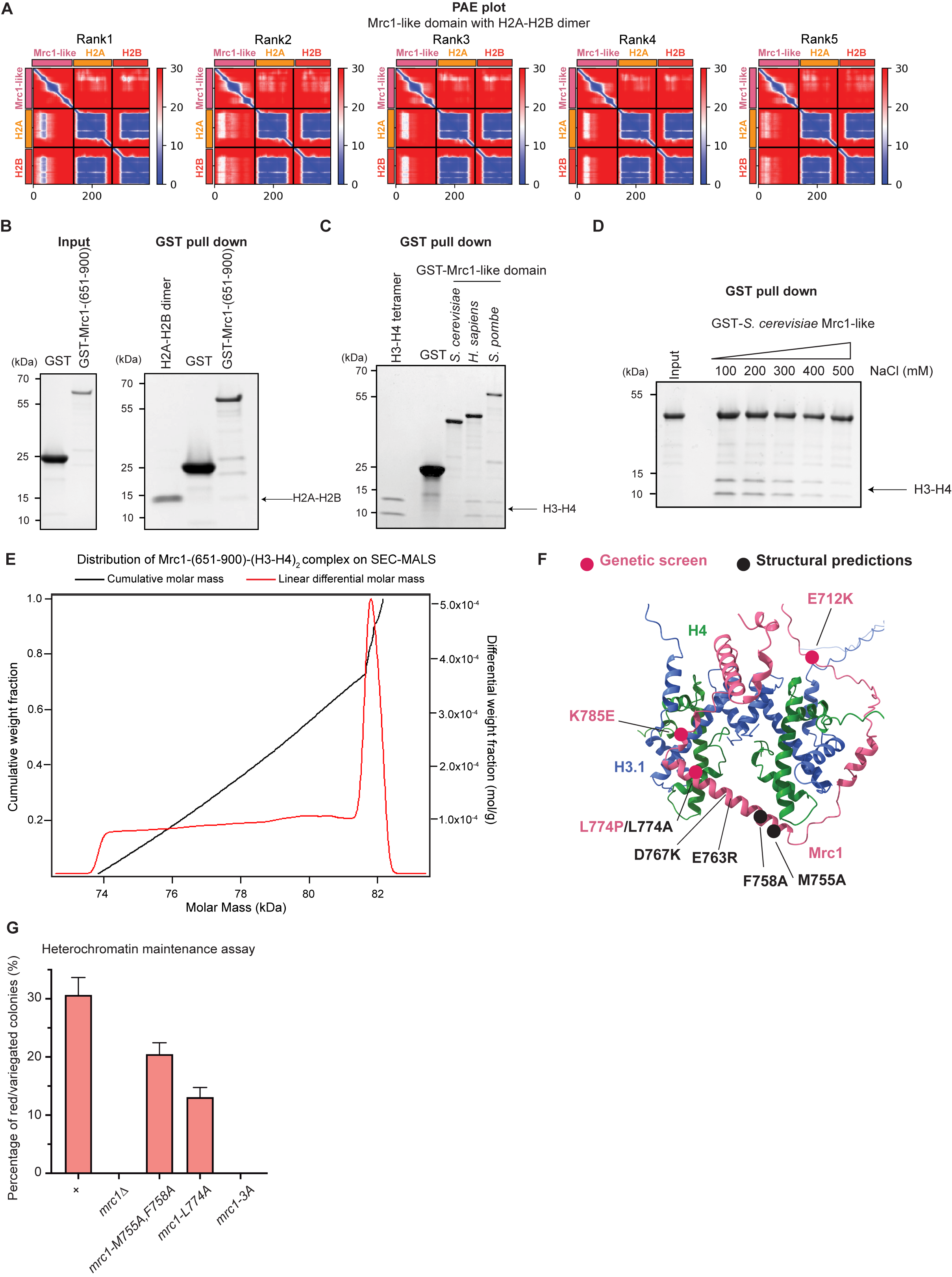

Supplement: 4 — Figure S4. Interactions between Mrc1-like domain and histones, related to Figure 3. A) PAE plots of predicted interaction between S. pombe Mrc1-like domain and H2A/H2B dimer. B) GST-Mrc1-like domain protein from S. pombe binds H2A-H2B weakly under stringent binding conditions (500 mM NaCl). C) GST-Mrc1-like domain fusion proteins from S. pombe and human pull down histone H3-H4 under stringent binding conditions (500 mM NaCl). D) GST pull-down assays showing that the interaction between S. cerevisiae Mrc1-like domain and H3-H4 is salt-sensitive. E) SEC-MALS distribution of Mrc1-(651–900)-(H3-H4)2 complex. Black curve indicates cumulative molar mass in the range of the indicated molar mass, and red curve indicates linear differential molar mass at the indicated molar mass. F) Diagram summarizing mutations of the Mrc1-like domain that specifically abolish heterochromatin maintenance isolated from targeted mutagenesis or generated based on structural predictions. G) Bar plot showing the percentage of red or variegated cells that maintain heterochromatin in the indicated mrc1 mutant cells in panel E; mrc1-3A (mrc1-M755A,F758A,L774A). n=3. Error bars indicate the standard deviation. [file NIHMS2018534-supplement-4.tif]

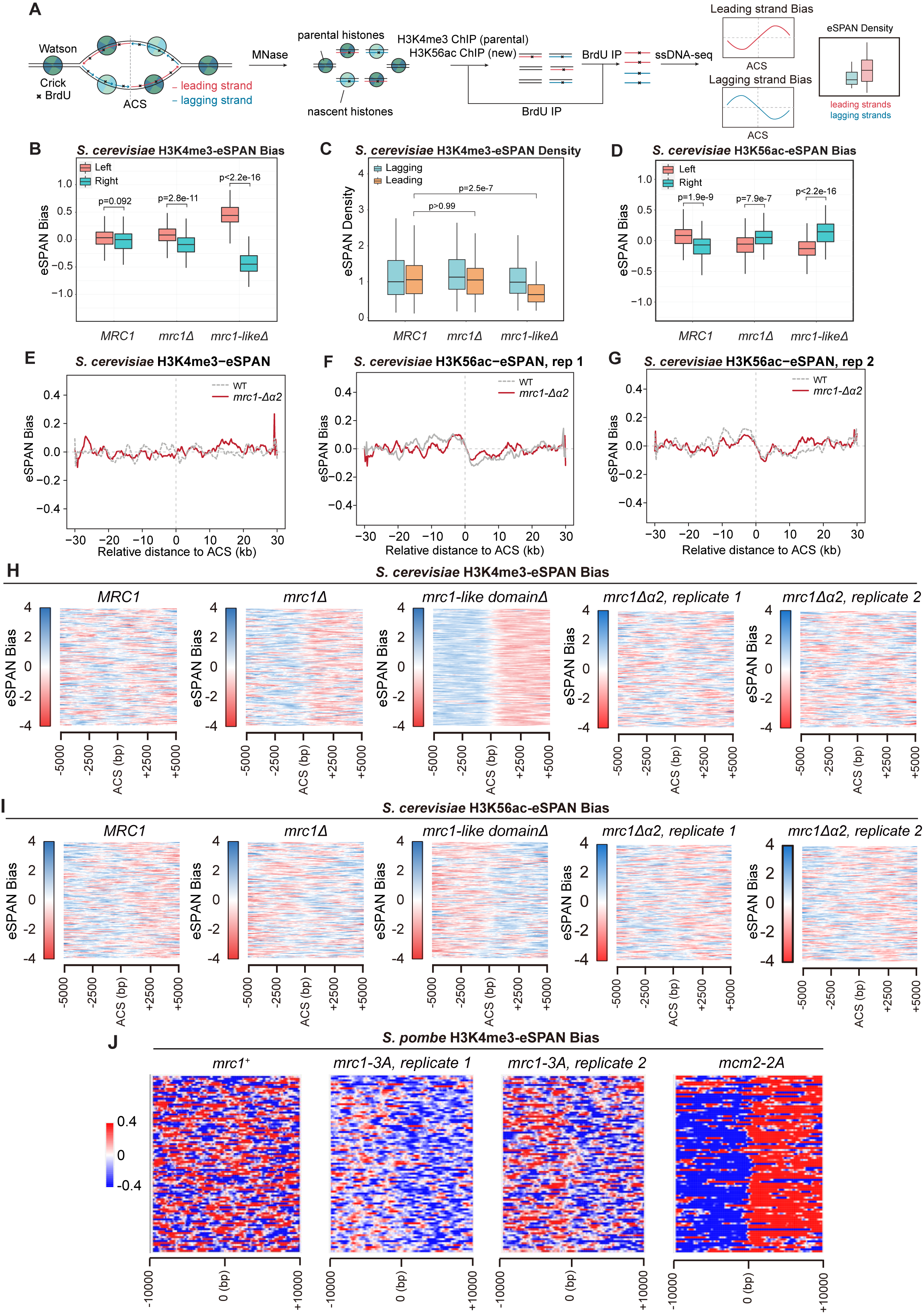

Supplement: 5 — Figure S5. Role of Mrc1 and its histone binding domain in parental histone transfer in S. cerevisiae and S. pombe, related to Figure 5. A) Diagram illustrates the workflow of eSPAN analysis of histone occupancy at nascent chromatin in S phase. The right panels illustrate the interpretation of eSPAN bias and the expected outcomes of leading or lagging strand biases of protein occupancy at the nascent chromatin. B) Bar plot showing the distribution of eSPAN bias of H3K4me3 between the left and right side of the 139 early ACS regions in the S. cerevisiae eSPAN samples shown in Figure 5D. P-values were determined by Wilcoxon rank-sum test. C) eSPAN density of parental histones (H3K4me3) at the leading strands and lagging strands in the S. cerevisiae eSPAN samples shown in Figure 5D. Statistical significance test at the leading strands between MRC1 and mrc1-ΔHBD cells: p = 2.5e-7, Wilcoxon rank-sum test. D) Bar plot showing the distribution of eSPAN bias of H3K56ac between the left and right side of the 139 early ACS regions in the S. cerevisiae eSPAN samples shown in Figure 4F. E) Second biological replicates of eSPAN bias of parental histone surrogate H3K4me3 in MRC1 wild-type, mrc1-α2Δ cells. F-G) Two biological replicates of eSPAN bias of new histone surrogate H3K56ac in MRC1 wild-type, mrc1-α2Δ cells H-I) Heatmap presentations of eSPAN bias of parental histones H3K4me3 (H) and newly deposited H3K56ac (I) among 139 early replicating ACSs in MRC1 wild-type, mrc1Δ, mrc1-like domainΔ (amino acid 711 to 850), and two replicates of mrc1-α2Δ S. cerevisiae cells. J) Heatmap presentations of eSPAN bias of parental histone (H3K4me3) among 162 early replicating ACSs in mrc1+, mrc1-3A (two biological replicates), and mcm2-2A S. pombe cells. [file NIHMS2018534-supplement-5.tif]

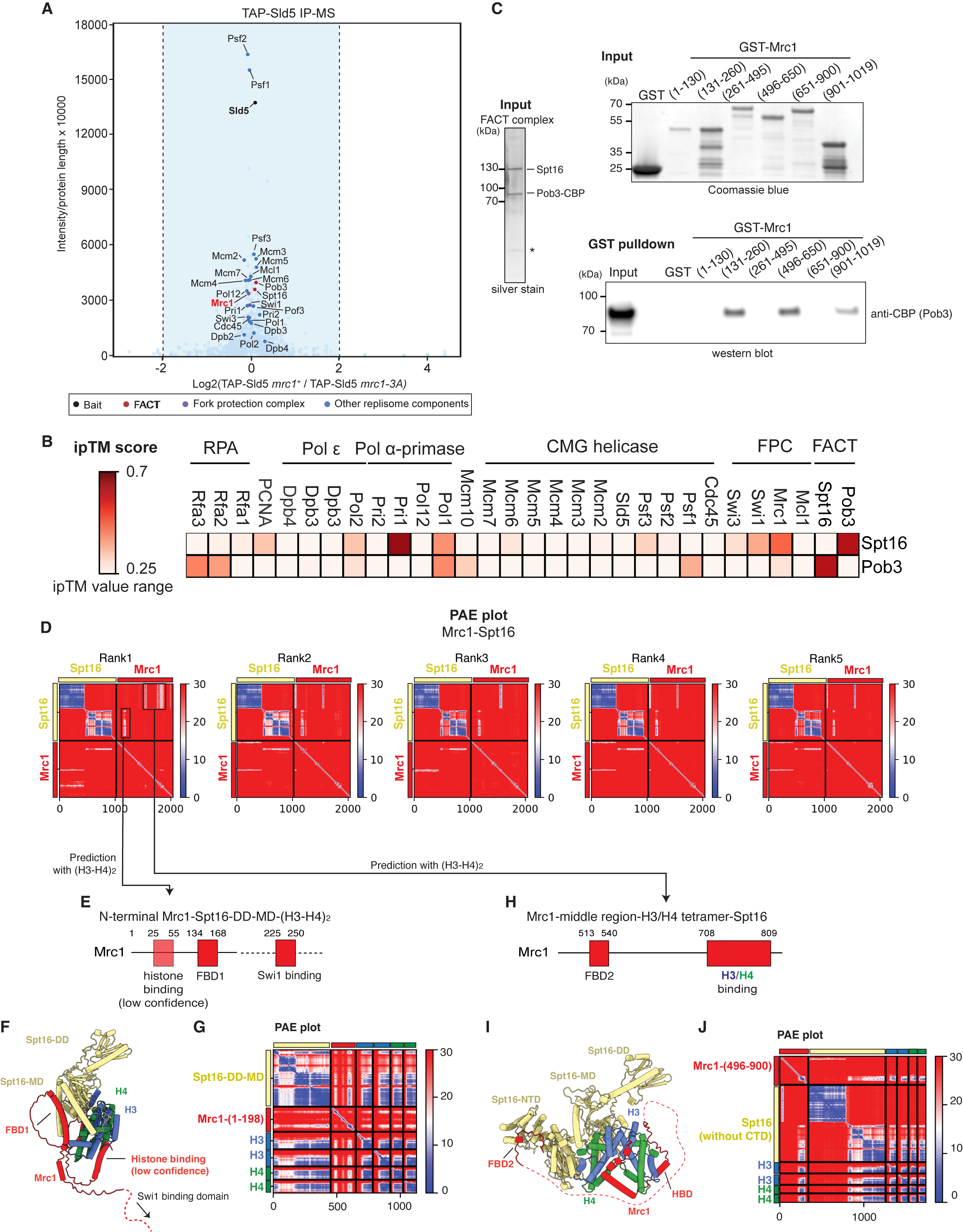

Supplement: 6 — Figure S6. Structural predictions suggest interactions between FACT and replisome components, related to Figure 6. A) IP-MS analysis of TAP-tagged Sld5 from mrc1+ and mrc1-3A cells. Colors indicate replisome components shown below the plot. B) Heatmap showing the average interface predicted template modeling (ipTM) score of all five predicted models between S. pombe FACT subunits and each core replisome component. The ipTM and heatmap scale ranges from 0.25 to 0.7. Although the ipTM score for the Spt16 and Pri1 interaction is high, the interaction interface appears to be small and clashes with the interaction interface between Pri1 and Pri2 in the published cryo-EM primase structure (PDB: 8B9C)104. C) In vitro GST pull-down assays using the indicated GST-Mrc1 segments to pull down endogenously purified FACT. SDS-PAGE gels show purified FACT (left), input (middle), and bound fractions (right). D) PAE of the AlphaFold-Multimer predicted interaction between Spt16-Mrc1. E) Based on the GST pull-down results, the first interaction between Mrc1 and FACT localizes at the N-terminus of Mrc1 (FBD1, amino acids 134–168) and the Spt16-middle domain. The location of other binding domains is shown for reference. F) the predicted structure of Mrc1-FBD1 in complex with Spt16-DD-MD and H3-H4 tetramer. G) The PAE plot of the predicted structure in panel F. H) The second interaction interface between Spt16 and Mrc1 localizes to a middle region of Mrc1 (Mrc1-FBD2, amino acids 513–540) and the N-terminal domain of Spt16 (Spt16-NTD). I) the predicted structure of Spt16 (without CTD)-Mrc1(middle region including FBD2)-H3-H4 tetramer. J) The PAE plot of the predicted structure in panel I. [file NIHMS2018534-supplement-6.tif]

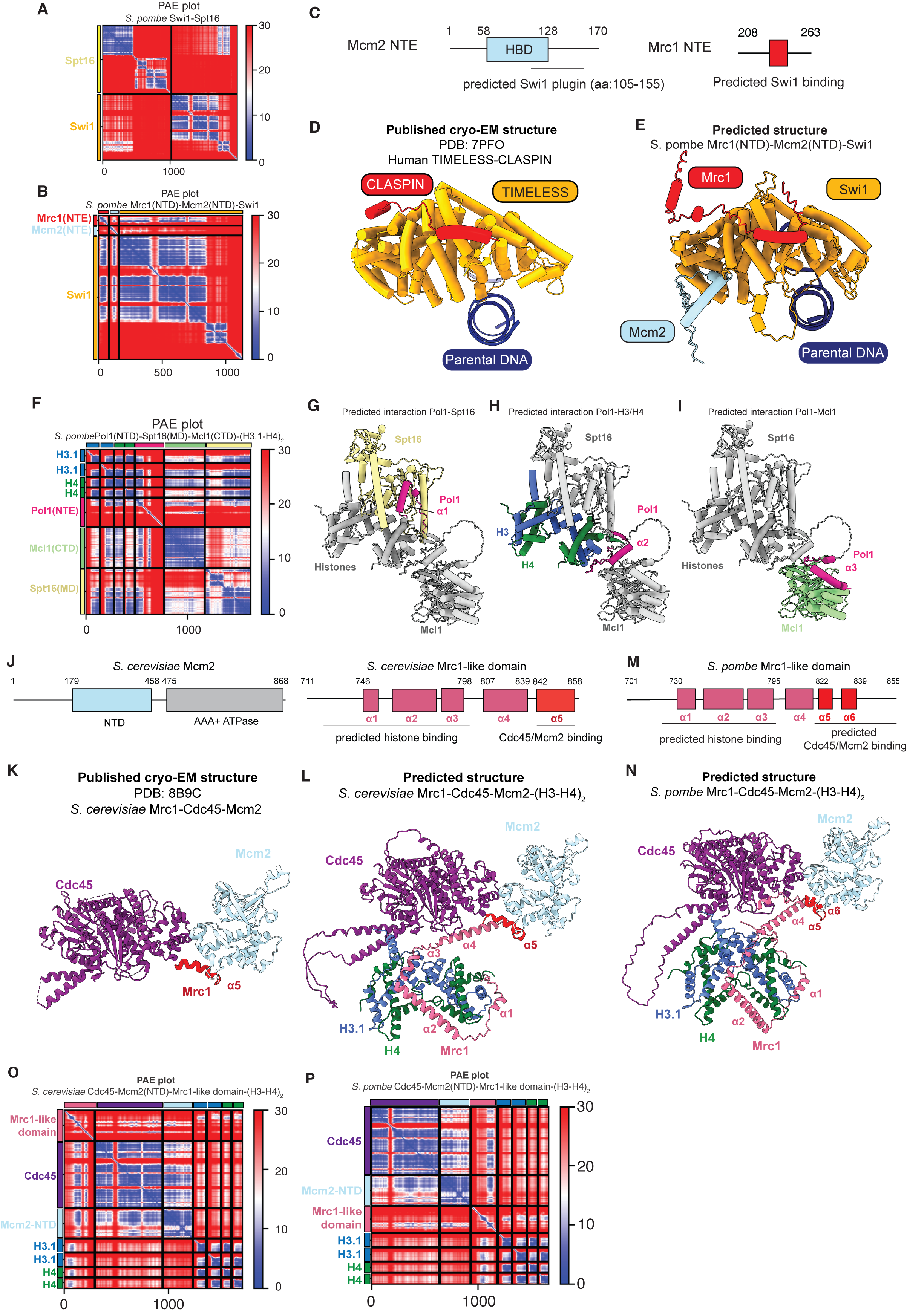

Supplement: 7 — Figure S7. Mapping the locations of FACT and parental histones on the cryo-EM structure of the replisome, related to Figure 6. A) PAE plot of the predicted structure of Swi1 and Spt16 interaction shown in Figure 7A–B. B) PAE plot of the predicted structure of S. pombe Mcm2(NTE)-Swi1(NTD)-Spt16(DD-MD)-(H3-H4)2 shown in Figure S7D. C) Summary of the NTEs of Mcm2 and Mrc1 predicted to interact with Swi1. D) Published cryo-EM structure (PDB: 7PFO) of human N-terminal CLASPIN bound to TIMELESS. E) Predicted structure of S. pombe N-terminal Mrc1 and Mcm2 bound to Swi1. F) PAE plot of the predicted structure shown in panel 6E. G) -I) Predicted structure in panel 6E with highlighted interaction between G) Spt16 and Pol1, H) H3-H4 and Pol1, I) Mcl1-CTD and Pol1. J) Diagrams indicating the domains of Cdc45, Mcm2 and Mrc1 shown in Figure S7K–L. K) Published cryo-EM structure (PDB: 8B9C) of S. cerevisiae Cdc45, N-terminal domain of Mcm2 and Mrc1-like domain interaction. L) Predicted structure of S. cerevisiae Mrc1-like domain with (H3.1-H4)2, Cdc45 and N-terminal domain of Mcm2. M) Diagram indicating the S. pombe Mrc1-like domain. N) Predicted structure of S. pombe Mrc1-like domain with (H3.1-H4)2, Cdc45 and N-terminal domain of Mcm2. O) PAE plot of the predicted structure shown in panel L. P) PAE plot of the predicted structure shown in panel N. [file NIHMS2018534-supplement-7.tif]

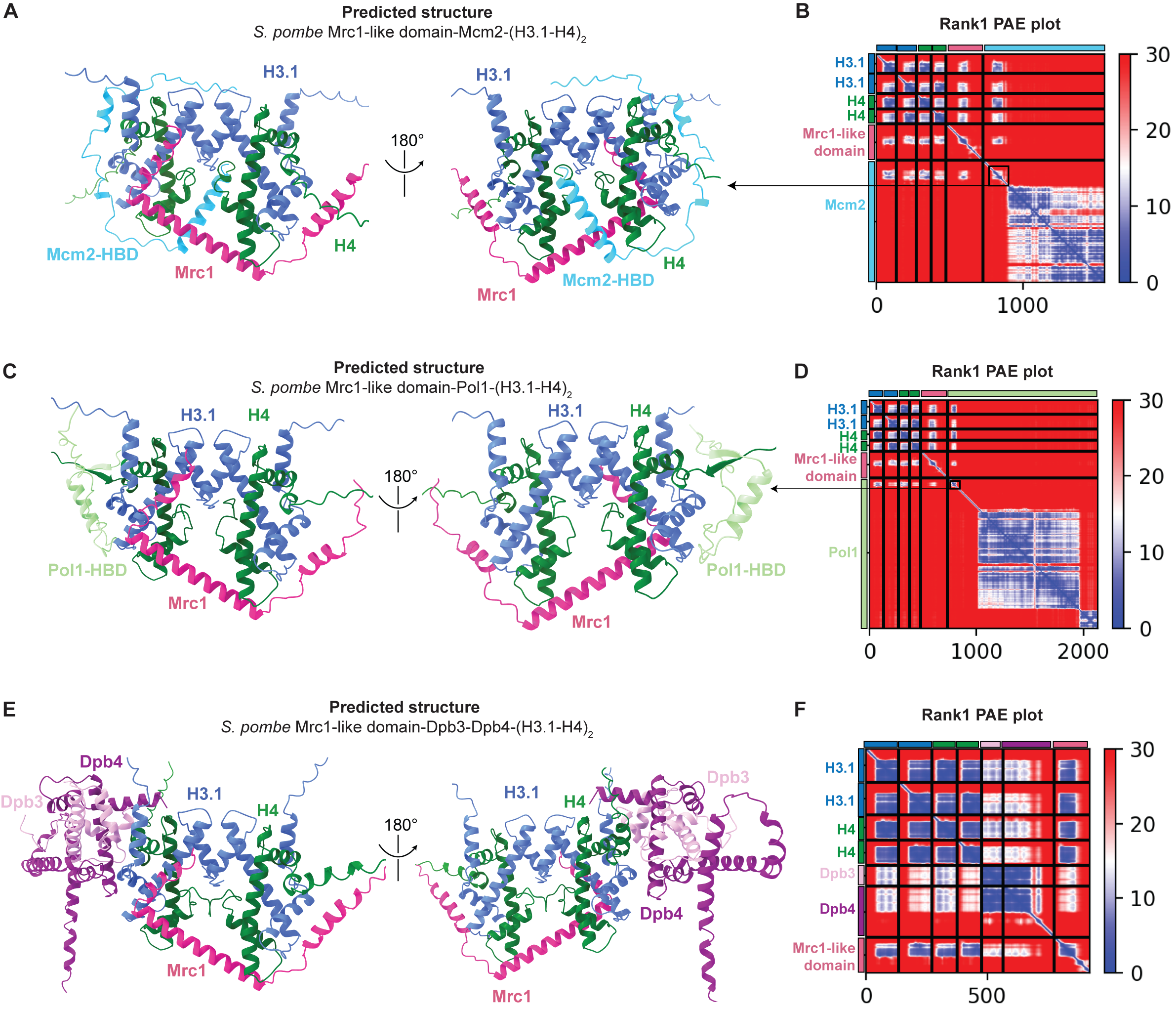

Supplement: 8 — Figure S8. Structural predictions suggest that the Mrc1 histone binding domain can bind H3-H4 tetramer together with other replisome histone binding domains, related to Figure 7. A) Predicted structure of S. pombe Mrc1-like domain with (H3.1-H4)2 and Mcm2. B) Rank 1 PAE plot of the predicted structure in panel A. C) Predicted structure of S. pombe Mrc1-like domain with (H3.1-H4)2 and Pol1. D) Rank 1 PAE plot of the predicted structure in panel C. E) Predicted structure of S. pombe Mrc1-like domain with (H3.1-H4)2 and Dpb3/Dpb4. F) Rank 1 PAE plot of the predicted structure in panel E. The confidence of the interaction between Dpb3-Dpb4 and (H3.1-H4)2 is lower compared to all other replisome histone binding components. [file NIHMS2018534-supplement-8.tif]
